# The Roles of Matrix Metalloproteinases and Their Inhibitors in Human Diseases

**DOI:** 10.3390/ijms21249739

**Published:** 2020-12-20

**Authors:** Griselda A Cabral-Pacheco, Idalia Garza-Veloz, Claudia Castruita-De la Rosa, Jesús M Ramirez-Acuña, Braulio A Perez-Romero, Jesús F Guerrero-Rodriguez, Nadia Martinez-Avila, Margarita L Martinez-Fierro

**Affiliations:** Molecular Medicine Laboratory, Unidad Académica de Medicina Humana y Ciencias de la Salud, Carretera Zacatecas-Guadalajara Km.6. Ejido la Escondida, Zacatecas 98160, Mexico; gris_elda_ai91@hotmail.com (G.AC.-P.); castruitade@gmail.com (C.C.-D.l.R.); jesusm.ra94@gmail.com (J.MR.-A.); alekhandroperes@gmail.com (B.AP.-R.); guerreroajf@gmail.com (J.FG.-R.); ik.otik88@gmail.com (N.M.-A.)

**Keywords:** matrix metalloproteinases, human diseases, metalloproteinase inhibitors

## Abstract

Matrix metalloproteinases (MMPs) are a family of zinc-dependent extracellular matrix (ECM) remodeling endopeptidases that have the capacity to degrade almost every component of the ECM. The degradation of the ECM is of great importance, since it is related to embryonic development and angiogenesis. It is also involved in cell repair and the remodeling of tissues. When the expression of MMPs is altered, it can generate the abnormal degradation of the ECM. This is the initial cause of the development of chronic degenerative diseases and vascular complications generated by diabetes. In addition, this process has an association with neurodegeneration and cancer progression. Within the ECM, the tissue inhibitors of MMPs (TIMPs) inhibit the proteolytic activity of MMPs. TIMPs are important regulators of ECM turnover, tissue remodeling, and cellular behavior. Therefore, TIMPs (similar to MMPs) modulate angiogenesis, cell proliferation, and apoptosis. An interruption in the balance between MMPs and TIMPs has been implicated in the pathophysiology and progression of several diseases. This review focuses on the participation of both MMPs (e.g., MMP-2 and MMP-9) and TIMPs (e.g., TIMP-1 and TIMP-3) in physiological processes and on how their abnormal regulation is associated with human diseases. The inclusion of current strategies and mechanisms of MMP inhibition in the development of new therapies targeting MMPs was also considered.

## 1. Introduction

The extracellular matrix (ECM) not only plays a supporting role for organs and tissues but also actively participates in other functions, such as regulation of the cell cycle and cell motility, survival, and apoptosis, as well as the distribution of growth factors and integration of signals into cells. The ECM is made up of hundreds of molecules, including proteoglycans; glycosaminoglycans; structural proteins, such as collagen and elastin; adhesion proteins, such as fibronectin and laminin; and proteases called matrix metalloproteases (MMPs) [1]. The MMPs belong to a family of endopeptidases that contains 23 members. These contain zinc, are dependent on calcium, and can degrade and remodel the proteins that form the ECM. They also participate in different biological and physiological processes that are regulated by hormones, growth factors, and cytokines [2]. Based on their sub-cellular distribution and specificity for components of the ECM, the MMPs are divided into membrane-type matrix metalloproteases (MT-MMPs), collagenases, gelatinases, stromelysins, and matrilysins (Table 1). Collagenases (MMP-1, MMP-8, MMP-13, and MMP-18) degrade triple-helical fibrillar collagen, which is fundamental in bone and ligaments. Gelatinases (MMP-2 and MMP-9) are involved in different cellular process including angiogenesis and neurogenesis; these proteases alter the molecules of the basal lamina, subsequently leading to cell death. Stromelysins (MMP-3, MMP-10, and MMP-11) are small proteases that degrade segments of the ECM. Matrilysins (MMP-7 and MMP-26) process cell surface molecules and digest ECM components. MT-MMPs have collagenolytic activity and may activate some proteases and components of the cell surface (Table 1) [1,3]. MMPs are also classified into eight groups according to their structure. Among these, five are secreted and three are bound to membranes (MT-MMPs) [1]. Some human MMPs display a signal peptide that directs them to the endoplasmic reticulum, the pro-domain (a pro-peptide with a thiol group that interacts with zinc and keeps them as inactive zymogens), and a catalytic domain with a zinc-binding site [1,2]. MMP-23 undergoes type II secretion and therefore does not have an N-terminal signal sequence. Despite most MMPs having N-terminal signal sequences, these do not result in 100% of the protease being secreted; signal sequences can be inefficiently recognized by the sec61 translocon, resulting in a significant fraction of the proteins remaining in the cytoplasm [4,5]. The conservation of an inefficient signal sequence in MMP-2 orthologs suggests the presence of selective pressure to retain a significant fraction of this protease within the cytoplasm. This could support the notion that MMP-2 has important, but unknown, physiological functions within cells. Table 1 displays the classification of MMPs according to their structures and substrates.

MMPs are inhibited by tissue inhibitors of MMPs (TIMPs), which are endogenous protein regulators. The TIMP family (TIMP-1–4), are proteins made up of 184–194 amino acids that are ≈21 kDa in molecular weight. The TIMP family has similar but not identical protease inhibitory profiles [8]. TIMPs are present in the ECM in a soluble form, except for TIMP-3, which is bound to the ECM. All TIMPs inhibit MMPs through reversible blockage, forming 1:1 stoichiometric complexes [8]. TIMPs selectively inhibit different MMPs and members of the families Disintegrin and Metalloproteinase (ADAM) and Disintegrin and Metalloproteinase with Thrombospondin motifs (ADAMTS) [8,9]. TIMPs also are important for the activation and uptake/removal of MMPs from the extracellular environment. TIMP function determines the influence of the ECM on cell phenotype, cell adhesion molecules, cytokines, chemokines, and growth factors. They are formed by an amino-terminal domain, which is the inhibiting domain that binds to the active site of MMPs and domain C. The ability of TIMPs to inhibit MMPs is due to their interaction in the terminal N-domain. Domain C gives TIMPs the ability to interact with the hemopexin domain of some MMPs [10]. In the next paragraphs, the expression, activity, regulation, and participation of MMPs and TIMPs in normal physiological processes are summarized, and their abnormal expression is associated with human diseases is detailed.

## 2. Roles and Expression of MMPs and TIMPs under Normal Physiological Conditions

MMPs play an important role in tissue remodeling during various physiological processes, such as embryogenesis, morphogenesis, angiogenesis, and wound repair. During normal biological processes, such as pregnancy and wound healing, alterations in MMP expression and activity occur [1]. MMPs and TIMPs are of great importance in the morphogenesis of fetal organs and subsequent events, having roles such as increasing the activity of cardiac morphogenesis [1] and lung differentiation as well as events necessary for the culmination of pregnancy and childbirth (Table 2) [2].

An example of the dynamic participation between MMPs and TIMPs occurs during lung differentiation and specialization, which occurs as a multistep process starting with branching morphogenesis (7–16 weeks of gestation (WG)), angiogenesis (16–24 WG), and alveolar generation (36 WG to 3 years old) [11]. Throughout morphogenesis, MMP-1, MMP-9, TIMP-1, TIMP-2, and TIMP-3 are detected in the fetal epithelium, while MMP-1, MMP-2, TIMP-2, and TIMP-3 are only expressed in the pulmonary vascular endothelium and media [11,22]. Afterwards, the canalicular stage is peculiar with regard to its angiogenesis and the linking of bronchiolar structures with their capillary interface. The last phase, the alveolarization stage, is characterized by alveolar development and maturation of the capillary network. This process requires MMP-14 and the coordination of extracellular matrix remodeling with epithelial morphogenesis and capillary growth [11].

After 20 WG, the main events that occur are substantial growth and weight gain. A considerable amount of subcutaneous fat appears with the formation of adipocytes from a precursor that involves two classifications: white adipose tissue (WAT) and brown adipose tissue (BAT). WAT is present in adults, while BAT is predominantly responsible for shivering thermogenesis in postnatal babies. [23]. Both of them require key transcription factors that are necessary to promote the differentiation of preadipocytes into mature adipocytes. The increased secretion of both MMP-2 and MMP-9 [24] was demonstrated through Western blot analysis and gelatin zymography. The results showed greater expression of MMP-2, MMP-9, and TIMP-2 by adipocytes. The amount of active MMP-2 increased as adipocytes differentiated [15,25]. On the other hand, the overexpression of TIMP-1 has been shown to result in enhanced involutional adipogenesis [16]. In addition, MMP-3 acts to slow down the adipogenic process [26]. MMP-11 plays a role in whole body metabolism and energy homeostasis, and it is a potent negative regulator of adipogenesis [27,28]. Bone develops in two different ways: mesenchymal cells can directly differentiate into bone by the process of intramembranous ossification (craniofacial skeleton) or it can differentiate into cartilage (endochondral ossification) and gradually turn into bone [17], with the latter being a process that occurs from the embryonic stage through to adulthood. One of the main MMPs implicated in endochondral ossification is MMP-13, which is derived from chondrocytes, synovial cells, and osteoblasts, and is considered the most important collagenase for the degradation of cartilage. It is induced by hypertrophic cartilage, and it plays a role in the degradation of type II collagen [19]. There are some secondary MMPs that also play important roles in this process, including MMP-2, MMP-9, MMP-14, and MMP-16. All of these are regulated at several levels, including gene expression, spatial localization, zymogen activation, and inhibition by naturally occurring inhibitors (TIMP-1–4), which are also expressed during endochondral ossification and osteocytic differentiation during bone matrix mineralization [19]. These kinds of regulatory events are described in the following sections.

### Normal Expression of MMPs and TIMPs in Adult Tissues

Metalloproteinases and TIMPs are normally expressed in various tissues. However, it has been described that during the development of different human diseases, metalloproteinases 1, 2, 3, 7, 8, 9, 13, and 14 are overexpressed in specific tissues. These tissues include the kidneys, liver, colon, placenta, intestines, stomach, bladder, pancreas, ovary, uterus, and bone marrow, among others. TIMPs have variable expression in specific tissues such as the breast, brain, lungs, liver, kidneys, colon, skin, ovaries, and heart. In these tissues, TIMP-1 is overexpressed, TIMP-3 is underexpressed, and TIMP-2 and TIMP-4 can be either underexpressed or overexpressed (Table 3 and Tables S1–S3).

## 3. Regulation of MMPs and TIMPs

**Figure 1:**
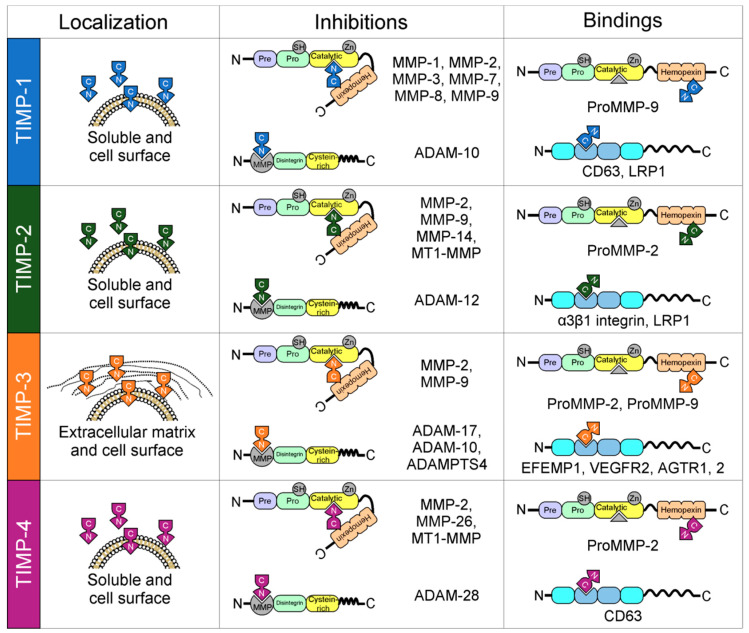
Localization and interactions between tissue inhibitors of matrix metalloproteases (TIMPs) and matrix metalloproteases (MMPs). All TIMPs are secreted, but only TIMP-3 is incorporated into the matrix. Structurally, TIMPs are comprised of two domains that pack side-by-side (N-terminal and C-terminal domains). The N-terminal domain is sometimes referred to as the “inhibitory domain”. TIMP-1 inhibits MMP-1–3 and MMP-7–9; TIMP-2 inhibits MMP-2, MMP-9, MMP-14, and membrane-type matrix metalloproteases 1 (MT1-MMP); TIMP-3 inhibits MMP-2 and MMP-9; and finally, TIMP-4 inhibits MMP-2, MMP-26, and MT1-MMP. In addition, TIMPs interact with the proforms of MMPs in a non-inhibitory manner, and they also have functions independent of MMP inhibition by directly binding to cell surface receptors (TIMP-1 to CD63; TIMP-2 to α3β1integrin and LRP1; TIMP-3 to EFEMP1, VEGFR2 and AGTR1,2; and TIMP-4 to CD63).

Under normal physiological conditions, the activity of MMPs is precisely regulated at four levels: (1) transcription, (2) activation of zymogen precursors, (3) interaction with specific components of the ECM, and (4) inhibition by TIMPs [78] (Figure 1). The activity of MMPs is inhibited by TIMPs through reversible blockage, and MMPs are expressed in specific tissues. TIMPs are comprised of two domains that pack side-by-side (N-terminal and C-terminal domains). The N-terminal domain is sometimes referred to as the “inhibitory domain”. TIMPs also have functions independent of MMP inhibition, whereby they directly bind to cell surface receptors. TIMP-1, which is secreted by the majority of the body’s cells, is more restricted in its inhibitory range than the other three TIMPs. It inhibits all types of MMPs (binding particularly strongly to MMP-9 and pro-MMP-9), with the exceptions of MMP-14, MMP-16, MMP-18, MMP-19 [79], MT1-MMP, MT2-MMP, MT3-MMP, and MT5-MMP [79]. TIMP-2 is expressed constitutively in most tissues, but it is not inducible by growth factors. TIMP-3 is expressed in tissues as a matrix protein and in the basal membranes of the eyes and kidneys, whereas TIMP-4 is expressed in the heart, ovaries, kidneys, pancreas, colon, testes, brain, and adipose tissue. In their specific tissues, TIMPs show specific expression in a constitutive or inducible manner, which is regulated at the transcriptional level by cytokines, growth factors, and chemokines [80,81].

There are some subtle differences between the affinities of TIMPs for other MMPs. For example, TIMP-2 and TIMP-3 inhibit MMP-3 and MMP-7 to a lesser extent than TIMP-1 does, which contrasts with their affinities for other MMPs [82]. TIMP-3 is unique among the mammalian TIMPs, as it inhibits a broader array of MMPs, as well as inhibiting several members of the ADAM and ADAMTS families [9]. TIMPs are also multifunctional proteins with pleiotropic activities mediated through MMP-independent protein–protein interactions [8]. The positioning of TIMPs at the surface of the cell (TIMP-2 and TIMP-3), in the matrix (TIMP-3), and as soluble forms (TIMP-1, TIMP-2, and TIMP-4) makes them versatile signal regulators [8]. TIMPs also interact with proforms (zymogen forms) of MMPs but in a non-inhibitory manner: TIMP-1 and TIMP-3 interact with pro-MMP-9, and TIMP-2 and TIMP-4 interact with pro-MMP-2. In these cases, only the C terminus is involved, leaving the N terminus free to associate with a second MMP molecule [10,83]. TIMPs are also involved in MMP activation and possible modulation through mediating interactions of active MMPs with specific substrates [84]. In vivo studies using MMP inhibitors and MMP knockout mice indicate that MMPs have essential roles in infection and host defense [85]. The activity of MMPs is mainly inhibited by TIMPs and α2-macroglobulin, in addition to their regulation at the transcription level by ECM components.

Is important to note that there is a fifth level of regulation that is not widely reported, and it involves the regulated absorption/elimination of active proteases from the extracellular environment. The vast majority of the literature is based on an analysis of changes at the transcriptional level (level 1), which provides inadequate information on the regulation and biologically relevant activities of proteases that are secreted and activated post-translationally. Our lack of knowledge about the regulation of MMP activity at the post-transcriptional level, particularly in vivo, is an important point that we must consider in future research.

## 4. MMPs and TIMPs in Human Diseases

The roles of MMPs and TIMPs in the maintenance of health and disease have gained interest. To understand how the regulation of these molecules plays a role in pathological conditions and how these molecules intervene in the secretion of growth factors, reactive oxygen species (ROS) and cytokines could be used to help to identify tools for better management of disease. Abnormal regulation of MMPs and TIMPs has a relevant role in pathological conditions, including inflammation, tissue destruction, fibrosis, abnormal angiogenesis, weakening of the matrix, microglial activation, autoimmune diseases, and carcinogenesis [86]. These compounds are involved in processes that include adhesion, cell proliferation, and migration and/or apoptosis by causing the cutting of bioactive molecules that modulate these processes [83]. The behaviors of MMPs and TIMPs in different sets of chronic diseases are described below.

### 4.1. MMPs and TIMPs in Diabetes Mellitus

Globally, the number of people with diabetes mellitus (DM) has increased in the past three decades, and DM is within the top ten causes of death [87]. The prevalence of DM varies among different populations with a greater incidence of DM in individuals with a higher native American admixture [88]. Type 2 DM (T2DM) has a strong genetic component—if both parents have T2DM, the risk of an individual developing T2DM increases by 40%. The disease is polygenic and multifactorial [89]. Insulin resistance, impaired insulin secretion, abnormal fat metabolism, excessive hepatic glucose production, and systemic low-grade chronic inflammation characterize T2DM. As insulin resistance and compensatory hyperinsulinemia progress, it is more difficult to sustain the hyperinsulinemic state [90]. DM is a disease that is considered a risk factor for cardiovascular disease (CVD) [91]. Hyperglycemia is one of the main feature of the disease, and it generates damage to the vascular system, nerves, eyes, kidneys, and heart [92]. MMPs and TIMPs are often regulated as a means to control excess MMP activity in DM. In patients with DM, constant hyperglycemia generates oxidative stress (OS). The synthesis of MMP-9 is induced by sustained hyperglycemia. This was demonstrated at the protein level, as the expression and activity of MMP-9 increased as a consequence of the oxidative stress generated in vascular endothelial cells [93]. TIMP-1 can mitigate the death of β cells in type 1 DM, because it enhances the replication of pancreatic islet β cells. Thus, the TIMP-1 gene may be a potential target in the prevention, or even reverseal, of type 1 DM [94]. However, TIMP-1 has also been associated with low-grade chronic inflammation in the adipose tissue of patients with T2DM [95]. Individuals with T2DM in combination with arterial hypertension exhibit maximum TIMP-1 levels and TIMP-1:MMP-2 and TIMP-1:MMP-9 ratios, as well as enhanced secretion of tumor necrosis factor alpha (TNF-α), interleukin-16 (IL-6), and IL-17 [96]. TIMP-3 has also been implicated in the pathogenesis of DM and vascular inflammation [69]. TIMP-3 is unique among TIMPs, because it retains its ability to inhibit shedding enzymes, such as ADAM17, which are involved in inflammatory processes. In atherosclerotic plaques from subjects with T2DM, the deregulation of ADAM17 and MMP-9 activities is related to the inadequate expression of TIMP-3 via SirT1 [97]. There is increasing evidence of the roles of MMP-2, MMP-9, MMP-11, MMP-13, and TIMP-4 in DM and metabolic disorders. In vivo studies have shown that the inhibition of MMP-13 may be beneficial for the treatment of human diabetic neuropathy [98,99], whereas MMP-11 overexpression is protective against T2DM [27]. The concentrations and activity of MMP-2 and MMP-9 are increased in the urine of patients with T1DM and T2DM, especially in patients with albuminuria and established renal injury [100]. MMP-2 is strongly induced in the adipose tissue of obese patients [101] and underlies the pathogenesis of diabetic cardiomyopathy by increasing the extracellular collagen content [102]. MMP-9 mediates diabetes-induced retinal neuropathy and vasculopathy [103] and is associated with the severity of diabetic retinopathy [104]. The absence of TIMP-4 ameliorates high-fat diet induced obesity due to defective lipid absorption in vivo [105]. Therefore, MMP-2, MMP-9, MMP-11, MMP-13, and TIMP-4 could be important biomarkers to evaluate in diabetes and associated disorders.

The ECM of the colon mucosa of patients with DM presents high levels of fibrillar collagen (types I and III) and fibronectin with an imbalance between the activities of MMPs and TIMPs and deregulation of the transforming growth factor beta 1 (TGF-β1) pathway associated with the appearance of myofibroblasts and the accumulation of ECM. The TGF-β1/Smad pathway plays a key role in the remodeling of intestinal tissue in DM [106]. On the other hand, in patients with vascular complications (including DM), the number of extracellular vesicles (EVs) significantly increases during the acute phase of the disease. EVs appear to be the upregulated cytokine transporters and angiogenic agents in patients with DM. The number of EVs is strongly influenced by the duration of the disease and successful treatment. There is evidence of increases in TIMP-1 and TIMP-2 in the EVs of patients with DM [107].

#### 4.1.1. Gestational Diabetes Mellitus

Gestational diabetes mellitus (GDM) has significant implications on the future health of the mother. Some clinical studies have suggested that subclinical inflammation and vascular dysfunction occur after GDM [51]. Studies show that during GDM, MMP concentrations increase, and after GDM, TIMP-1 can suppress the levels of MMP-8 and MMP-9. TIMP-1 exerts MMP-independent actions, such as pro-inflammatory and growth-factor-like properties, that contribute to low-grade inflammation [51]. The abnormal expression of MMPs and TIMPs modifies the ECM during vascular remodeling, causing dysregulated angiogenesis in patients with DM [108,109]. This abnormal regulation has been associated with the development and progression of diabetic microvascular complications, such as nephropathy, cardiomyopathy, retinopathy, and peripheral neuropathy [110]. In the case of diabetic wounds, MMP-1, MMP-2, and MMP-9 have roles in the re-epithelialization of wounds through the migration of keratinocytes (which is further explained below) [29], and the hypoxic and inflammatory environment of wounds of patients with DM is accompanied by an increase in reactive oxygen species (ROS) and an overproduction of MMP-9, which generates damage to the tissue and leads to poor wound healing [111]. MicroRNAs (miRs) play important roles in the initiation and progression of many pathological processes, such as diabetic nephropathy [69] and retinopathy [112]. *TIMP-3* is a target of miR-365 and is negatively regulated by it. miR-365 is highly expressed in the retina, and the dysfunction of the miR-365/TIMP-3 pathway is closely related to diabetic retinopathy. The mechanism by which this occurs could involve oxidative stress. When miR-365 is inhibited, the expression of TIMP-3 is upregulated. The miR-365/TIMP-3 pathway is a potential therapeutic target for the treatment of diabetic retinopathy [112].

#### 4.1.2. Diabetic Nephropathy

Diabetic nephropathy is the leading cause of chronic kidney disease (CKD) and end-stage renal disease, requiring renal replacement therapy. Patients with kidney disease predominantly account for the increased mortality rate observed in T2DM [113]. The pathogenesis of diabetic nephropathy is related to chronic hyperglycemia which, in turn, is associated with increased ROS production in multiple organs, including the kidneys [114]. Some known factors involve structural changes in the glomerulus (basement membrane thickening, mesangial expansion, fibrosis, and increased extracellular matrix), the effects of soluble factors (angiotensin II, growth factors, advanced glycation end products, and endothelin), and hemodynamic alterations in the renal microcirculation (increased glomerular capillary pressure, hyperperfusion, or glomerular hyperfiltration) [89].

In the DM population, the expression of MMP-2 and MMP-9 is altered, which contributes to microangiopathic and macroangiopathic complications, such as nephropathy, with MMP-2 being a good index of microangiopathy severity and MMP-9 being a good marker of macroangiopathy. In ex vivo models, at the protein level, MMP-9 has shown activity in endothelial cells and regulation by high concentrations of glucose [38]. Macrophages infiltrating the glomeruli secrete MMPs, leading to an imbalance between ECM degradation and renewal, which results in proteinuria and renal failure [115]. Animal models of diabetes have shown an inhibition of MMP proteolytic activity in renal tissues [116]. The accumulation of ECM characteristic of diabetic nephropathy is partially caused by the profibrotic proteins TGF-β and connective tissue growth factor. ADAM17 and its inhibitor, TIMP-3, are involved in nephropathy, and the loss of TIMP-3 underlies the development of diabetic nephropathy via FoxO1/STAT1 interplay [69]. It was originally thought that MMPs antagonized the development of fibrotic diseases. It has also been reported that MMP-9 plays a role in atherosclerosis development, and both MMP-2 and MMP-9, along with growth factors and cytokines, play roles in the development of proteinuria, tubulointerstitial fibrosis, and kidney disease progression [117]. MMP-2 has been related to the pathogenesis of chronic kidney disease, and its increased expression generates kidney damage as a result of ischemia–reperfusion injury. MMP-2 leads to structural alterations at the level of the tubular basement membrane and can generate all of the common features of kidney disease, including glomerulosclerosis, tubular atrophy, and interstitial fibrosis [118]. In terms of epigenetic regulation, it has been shown that hypomethylation of the *TIMP-2* gene can be associated with albuminuria in patients with early diabetic nephropathy [119]. MicroRNAs play important roles in the initiation and progression of many pathologic processes. miR-21 contributes to renal fibrosis mediated by MMP-9/TIMP-1 and improves the glomerular lesions induced by TGF-β and hyperglycemia through the repression of proapoptotic signals, which inhibits the loss of podocytes [120]. It has been proposed that miR-21 also regulates TIMP-3. According to the above discussion, the inhibition of miR-21 may be a new target for diabetic nephropathy [120].

### 4.2. MMPs and Wound Healing

Wound healing is a process carried out by re-epithelialization and is based on two functions: proliferation and cell migration [121]. The process of wound healing is governed by complex interactions between proteins and the ECM, involving a range of signaling pathways [122]. MMPs are involved in all wound-healing events. The functions of these are diverse and are not only involved in changes of the ECM. For example, during inflammation, neutrophils infiltrate the wound to protect against infection and release MMP-8, which is required for debridement of the wound and to cleave damaged collagen type I. In vivo studies show that MMP-8 deficiency leads to TGF-b signaling, inflammation, and delayed wound healing [123]. In injured skin, MMP-1 production is induced by keratinocytes that bind to type I collagen in the dermis through α2 and β1 integrins [30]. The proper functioning of MMP-1 is necessary for the migration of keratinocytes into type I collagen. This inductive response is controlled by the α2β1 integrin on migrating cells [124]. MMP-2 and MMP-9 play important roles in the cell migration involved in wound healing. Normally, MMP-2 participates in the proteolysis of laminin-5 during the phase of prolonged remodeling, which modulates the migration of keratinocytes to the final stage of wound healing [125]. MMP-1, MMP-2, and MMP-9 also contribute to wound re-epithelialization by loosening the tight contacts that keratinocytes initially establish with the dermal matrix. MMP-1 also takes part in the reduction of normal and hypertrophic scars [29]. The breaking of laminin-5 by MMP-2 promotes the cell migration of epidermal cells [126]. MMP-9 is upregulated by TGF-β through the activation of ligands of TGF-β and maintenance of the generation of keratinocyte migration [127].

In adiabetic patients, wound healing is known to require a balance between the accumulation of collagenous and non-collagenous ECM components and their remodeling by MMPs and TIMPs. MMP-8 and MMP-9 are involved in wound-healing events, and their physiological functions are the degradation of damaged type I collagen and the facilitation of keratinocyte migration. In addition, under the microenvironment of hypoxia and inflammation that is generated in diabetic foot ulcers (DFUs), the production of ROS increases, maintaining an upregulation of MMP-9, which damages the tissue and leads to poor wound healing [111]. MMP-8 also plays a role in the wound healing response, and MMP-9 is part of the physiopathology of DFUs [128]. New treatment strategies for healing chronic DFUs could be directed toward reducing concentrations of MMPs and increasing concentrations of TIMPs [129]. Compared with healing in normal patients, the combination of MMPs and decreased concentrations of TIMP-2 in chronic DFUs suggests that an increased proteolytic environment contributes to the failure of healing in the wounds of diabetics [129]. Infection is a major cause of diabetic foot syndrome, being aggravating by the increased burden of multi-resistant microorganisms. In DFU treatment, there is a decrease in the concentration of local IL-6, followed by a fall in MMP-9 and an increase in TIMP-1, resulting in better healing and a reduction in wound size [64]. For example, in a randomized, double-blind, placebo-controlled pilot study, the extract of Quercus robur (Robuvit^®^) considerably reduced oxidative stress and MMP-9 activity in patients after hysterectomy, improving the condition of the patients [130].

### 4.3. MMPs and TIMPs in Renal Pathologies

The expression of MMPs and TIMPs in the kidneys is complicated, and the patterns of expression have not been fully characterized. In the kidneys, MMP-2, MMP-3, MMP-9, MMP-13, MMP-14, MMP-24, MMP-25, MMP-27, MMP-28, TIMP-1, TIMP-2, and TIMP-3 are expressed [36]. In patients with renal disorders, an alteration of the ECM components of the kidneys is generated, leading to destruction of the parenchyma [131]. The role of MMPs in renal disease is the recruitment of inflammatory cells and chemotaxis, which regulates the inflammatory response. MMPs, along with TIMPs, regulate the activation, cytokine release, angiogenesis, chemotaxis, proliferation, and apoptosis of multiple inflammatory cells as well as the epithelial–mesenchymal transition, with the latter leading to the development of kidney fibrotic diseases [132]. The presence of MMP deregulation is implicated in renal physiopathological processes, both acute and chronic, such as acute kidney injury (AKI), glomerulosclerosis/tubulointerstitial fibrosis, diabetic nephropathy, polycystic kidney disease, and renal cell carcinoma [36]. In acute kidney injury, MMPs are suggested to play a role in the endothelial injury that is generated during ischemia–reperfusion. MMPs are involved in changes in the vascular endothelium, glomeruli, and tubular epithelial cells due to an increase in MMP-9 activity, which is associated with the degradation of endothelial cells and subsequent increased vascular permeability [133]. MMP-3 is considered an emerging therapeutic agent and a biomarker of kidney injury. In vitro studies have shown that it is upregulated by physiopathogical stimuli in the context of acute kidney injury [46]. The significance of MMPs and TIMPs in the progression of kidney pathologies has also been reported. The excess accumulation of the ECM is the main pathological mechanism of renal fibrosis [134,135]. TIMP-1 is the best predictor of TGF-β1, which is a hallmark of fibrosis [136], and it has been shown to be the best predictor of the presence of E-cadherin [137]. Several studies have shown elevated levels of MMP-2 and TIMP-2 in the serum of patients with chronic kidney disease (CKD) [37].

#### Glomerular Diseases

There are many forms of glomerular disease with variable routes of pathogenesis [138]. Glomerular epithelial/mesangial cells may express epitopes that mimic other immunogenic proteins made in the body. Microorganisms can infect the kidneys, producing their own antigens [139]. Amplification mediators (proteases and oxidants) expand this inflammation and, depending on the location of the target antigen of the host, basement membranes are damaged with either extracapillary or endocapillary proliferation, inducing mononuclear cell infiltration [140]. Macrophages, T-lymphocytes, and neutrophils are drawn into the glomerular tuft by chemokines, producing more proteases and cytokines that damage capillaries, the mesangium, and/or the glomerular basement membrane [89]. By themselves, mononuclear cells can injure the kidneys, as autoimmune events produce a humoral immune response [89]. Obstruction of the tubules with debris or by extrinsic compression functionally results in aglomerular nephrons. A second suggested mechanism is interstitial changes (fibrosis or interstitial edema) that alter vascular and tubular architecture and thereby compromise the normal tubular transport of water and solutes [141]. A third mechanism involves changes in vascular resistance due to damage to peritubular capillaries. Tubular cells are very metabolically active, and, as a result, decreased perfusion leads to tubular ischemic injury [89]. Then, the impairment of glomerular arteriolar outflow leads to increased intravascular hypertension in less involved glomeruli [139]. As well as the podocytes, other components of the glomerular filtration barrier are known to influence filtration [142].

In glomerular disease, MMPs are the main regulators of ECM degradation as well as the structural and functional integrity in the glomerulus, and when altered, they induce degradation of the architecture of the GBM [117]. The regulation of MMPs and TIMPs is considered to contribute to the maintenance of homeostasis in the production and degradation of the ECM in the glomeruli [36]. Glomerulosclerosis is one of the most frequent types of histological lesions associated with kidney disease. It emerges from an imbalance between the synthesis and degradation of the renal ECM [143]. MMP-2 and MMP-9 are expressed and secreted by glomerular cells, including mesangial cells and podocytes, and the function of these is to control the turnover of the glomerular basement membrane. Studies have emphasized the role of MMPs in the development or progression of the disease, which is possibly due to a decrease in the production of MMPs or an increase in the concentrations of their inhibitors. TIMP production serves as a significant mechanism for the post-translational modulation of MMP activity [143]. Disruption of the glomerular basement membrane via cytokine-induced alterations in MMPs and TIMPs is an important mechanism in the renal disease process. In renal disease, both infiltrating immune cells and resident renal cells are capable of secreting pro-inflammatory cytokines, including IL-1β and TNF-α [144]. Cell cycle arrest plays an role in the protection of renal tubular epithelial cells. G1 phase cell arrest serves as a protective mechanism following AKI, avoiding the replication of damaged DNA. TIMP-2 is associated with G1 cell cycle arrest during the very early phase of cellular damage and can serve as an biomarker to predict acute kidney injury in vivo [145]. Studies show that the cytokine TNF-α has different effects on the mesangial expression of MMP-9 and TIMP-1 through the following different signaling pathways: PKC, ERK ½, and p38 MAPK. This suggests that pro-inflammatory cytokines have an important role in the progression of kidney disease.

### 4.4. MMPs and TIMPs in Neurodegenerative Diseases

MMPs and TIMPs are involved in physiological processes such as neurogenesis, oligodendrogenesis, and cerebral plasticity [146]. They are essential for brain development due to their associations with neurophysiological functions. Furthermore, MMPs fulfill important functions in the central nervous system (CNS) during growth and development. They have important roles during the neuronal damage generated in acute and chronic conditions, as well as in neuronal repair processes [147,148]. In the CNS, the ECM includes proteoglycans involved in the development, survival, and activity of neuronal cells among its components. Neurodegenerative diseases are characterized by the death of neurons in different regions of the nervous system and consequent functional deterioration. MMP-2 and MMP-9 are expressed by neurons, astrocytes, and microglia in the CNS, and they play a physiopathological role in the disruption of the blood–brain barrier (BBB) through microglia activation and further progression of inflammatory cells [149]. The abnormal modulation of MMPs also participates in the pathogenesis of multiple sclerosis (MS), Alzheimer’s disease (AD), and Parkinson’s disease (PD) [131]. The inhibition of MMPs has been proposed as a possible therapeutic strategy for the treatment of neurodegenerative diseases [48]. The mechanisms of action by which MMPs contribute to the aggravation of neurodegenerative diseases are slowly starting to be elicited. It has been shown that MMPs participate in a common pathway related to the pathological changes in CNS homeostasis, that is, the accumulation of pro-inflammatory molecules or aggregates of proteins and peptides, leading to the increased permeability of the CNS barrier and, therefore, to cell death [48].

In an intriguing genetic correlation between TIMPs and the nervous system, three of the genes encoding TIMPs occur within introns of genes encoding synapisins. The *TIMP-1* gene resides within the *SYNAPSIN-1* gene, the *TIMP-3* gene resides within the *SYNAPSIN-3* gene, and the *TIMP-4* gene resides within the *SYNAPSIN-2* gene. TIMP-2 is expressed in post-mitotic neurons and promotes neurite outgrowth and the differentiation of cells [150] as a result of cell cycle arrest through increased production of the cyclin-dependent kinase inhibitor p21Cip and decreased expression of cyclins B and D. In in vitro models, TIMP-2 expression has been found in α3 integrin-positive cells, suggesting that TIMP2-α3β1 integrin interactions participate in neurogenesis [151]. TIMP-1 is considered to be a candidate gene related to plasticity, whose expression increases greatly in the hippocampus [152]. Studies show that the absence of TIMP-1 leads to significant deterioration in the formation and recall of reward associations [153]. This effect is probably mediated by an effect on neuronal development and not by the direct action of TIMP-1 on the memory.

#### 4.4.1. Multiple Sclerosis

Multiple sclerosis, also known as demyelinating myelopathy, is a disease characterized by the appearance of demyelinating, neurodegenerative, autoimmune, inflammatory, and chronic CNS lesions [48]. The disability seen in MS is related to axonal damage and the loss of neuronal cells. MS is approximately 3-fold more common in women. The age of onset is typically between 20 and 40 years of age [154]. Well-established risk factors for MS include a genetic predisposition, vitamin D deficiency, Epstein–Barr virus exposure after early childhood, and cigarette smoking [89].

The human leukocyte antigen (HLA) association, first described in the early 1970s, suggests that MS is an autoimmune disease [155]. The strongest susceptibility signal maps to the HLA-DRB1 gene in the class II region of the major histocompatibility complex (MHC), which accounts for ≈10% of the disease risk. The lesions that occur in MS begin with perivenular cuffing by inflammatory mononuclear cells, predominantly macrophages and T-lymphocytes, which infiltrate the surrounding white matter. At sites of inflammation, the BBB is disrupted, while the vessel wall is preserved [156]. The involvement of the humoral immune system is evident, and the complement is activated by myelin-specific autoantibodies that are present on degenerating myelin sheaths. Demyelination is a pathological hallmark and is found at the earliest time points of tissue injury [157].

Accordingly, in MS, the imbalance of MMPs and TIMPs cause a series of effects that cause blood–brain barrier breakdown and the infiltration of peripheral blood leukocytes, which is followed by degradation of the myelin and, subsequently, axonal disruption and neuronal cell loss [158]. Various brain and immune cells can secrete MMPs. The expression of MMP-7 and MMP-9 has been observed in blood vessels of active lesions, MMP-3 has been observed in endothelial cells, and MMP-1, MMP-2, MMP-3, and MMP-9 are found around active and necrotic lesions [48]. It has been proposed that MMP-9 is secreted by T-lymphocytes and macrophages, and its constant expression throughout the disease could be what contributes to the loss of neuronal cells and damage to the surrounding tissue [159]. MMP-9 increases the permeability of the BBB, facilitating the infiltration of leukocytes into the CNS, degrading the myelin layer, and consequently, generating neuronal damage [160]. As TIMP-1 is a tissue inhibitor of MMP-9, a considerable elevation in the MMP-9:TIMP-1 ratio has been observed in patients with MS [161]. Studies suggest that a change in the MMP-9:TIMP-1 ratio with respect to the proteolytic activity of MMP-9 may be the consequence of immune downregulation in MS [162]. In particular, the MMP-9:TIMP-1 ratio seems to be a very suitable and easily measurable biomarker for the continuous inflammation in MS and may be predictive of relapsing-remitting MS, which is detected by magnetic resonance imaging [163].

Elevated levels of MMP-9 in the serum of MS patients, together with increases in TIMP-1 and TIMP-2, have been observed in different studies [164]. TIMP-2 is also elevated in the monocytes of MS patients. The serum MMP-2:TIMP-2 ratio may represent a useful indicator for monitoring MS patients during the recovery phase [163]. MMP-2, MMP-14, and TIMP-2 could be considered interesting targets for potential therapeutic interventions due to their roles in the entry of monocytes to the CNS and the recovery of CNS lesions in patients with MS [60]. ADAMTS is inhibited by TIMP-3, and studies have shown the expression of ADAMTS (1, 4 and 5) and TIMP-3 in normal and MS CNS white matter, where ADAMTS4 mRNA increases and TIMP-3 decreases significantly in MS patients compared to controls [70]. Finally, MMP-12 is expressed in MS lesions at different stages of its evolution, and its expression has been demonstrated in chronic active demyelinating lesions [165].

#### 4.4.2. Parkinson’s Disease

Parkinson’s disease (PD) is the second most common age-related neurodegenerative disease, exceeded only by Alzheimer’s disease. The mean age of onset of PD is about 60 years old, and the lifetime risk for its development is ≈2% for men and 1.3% for women [89]. About 5–15% of cases are familial in origin, and mutations have been identified in several PD-linked genes. While monogenic mutations have been shown to be causative of PD, genetic risk factors that increase the risk of developing PD have also been identified [166].

Pathologically, the hallmark features of PD are reduced striatal dopamine, intraneuronal protein inclusions (Lewy bodies and Lewy neurites), and degeneration of dopaminergic neurons in the substantia nigra pars compacta [167]. Most cases of PD occur sporadically (≈85–90%) and are of unknown cause [89]. Several factors have been implicated in the pathogenesis of cell death in PD, including inflammation, excitotoxicity, oxidative stress, mitochondrial dysfunction, apoptosis, necrosis, the accumulation of misfolded proteins, and autophagic degeneration. Recent studies have shown that with aging, dopamine neurons are vulnerable to calcium-mediated neurotoxicity [168]. These neurons, along with innate immune cells residing in the CNS and microglia activation, produce factors such as interleukins, TNF-α, growth factors, substance P, reactive nitric species, ROS, nitric oxide, intracellular calcium elevation, activation of mitogen-activated protein kinases (MAPK), activation of nuclear factor kappa B, and MMPs [169]. These are pro-inflammatory factors that are toxic to neurons, since they lead to CNS disorders such as PD [47]. In PD, there is MMP overexpression, which generates damage at the neuronal level; this uncontrolled expression is only decreased by the TIMPs, as seen in PD, with increased expression of TIMP-1 and decreased expression of MMP-2 in the substantia nigra [39]. MMP-3 is produced by dopaminergic neurons stressed by neurotoxins, reproducing in a self-sufficient manner in the absence of inflammatory molecules, suggesting that it intervenes in the mechanism of apoptotic cells [47]. MMP-3 plays roles in apoptosis signaling and microglial activation [170]. It is known that MMP-3 influences the pathogenesis of PD, contributing to the loss of dopaminergic neurons [171]. Regarding the therapeutic opportunities related to MMP inhibition in PD, in vivo studies have shown the expression of TIMP-1 and TIMP-2 in the substantia nigra of postmortem brain samples of PD patients [39]. Other studies have shown increased levels of TIMP-1 in the cerebrospinal fluid (CSF) of patients with PD [172], as well as a protective effect of TIMP-1 polymorphisms in PD [173]. Therefore, MMP inhibitors may be promising for the management of PD, because the death of dopaminergic neurons seems to be related to the release of MMPs.

#### 4.4.3. Alzheimer’s Disease

Alzheimer’s disease, also called senile dementia of Alzheimer’s type, is the main cause of dementia and is a great healthcare challenge. Approximately 10% of all persons aged >70 years have significant memory loss, and in more than half of these people, the memory loss is caused by AD. Patients present with a loss in episodic memory, followed by slowly progressive dementia [89]. Brain atrophy is caused by the death of neuronal cells and a decrease in the presence of dendrites in the cerebral cortex and other subcortical areas. In addition, amyloid plaques and neurofibrillary tangles that are linked to cerebral atrophy are present. One of the characteristics that distinguishes this disease is the formation of abnormal amyloid-β (Aβ) oligomers and abnormally phosphorylated tau proteins that are added to the amyloid plaques, which could be the main cause of neuronal dysfunction [174]. Aβ is a protein derived proteolytically from a larger transmembrane protein. It is known as amyloid precursor protein (APP) [175]. The APP has neurotrophic and neuroprotective properties. The plaque core is surrounded by a halo, which contains dystrophic tau-immunoreactive neurites and activated microglia. The accumulation of Aβ in cerebral arterioles is termed amyloid angiopathy [176]. Neurofibrillary tangles are composed of neuronal cytoplasmic fibrils. Tau binds to and stabilizes microtubules, supporting the axonal transport of glycoproteins, organelles, and neurotransmitters throughout the neuron [177]. Once hyperphosphorylated, tau cannot bind properly to microtubules and redistributes from the axon throughout the neuronal cytoplasm and distal dendrites, compromising function. Other theories emphasize that abnormal conformations of tau induce the misfolding of native (unfolded) tau into pathological conformations and that this prion-like templating process is responsible for the spread of tau [178]. Excess production of Aβ42 oligomers is a key initiator of cellular damage in AD [89].

The remodeling of the pericellular environment by MMPs is regulated by modulation of their actions on neurotransmitters, growth factors, receptors, and other cell surface components, such as adhesion molecules. One of the factors opposing the ability of MMPs to maintain the stability of the structures is the degradation of the APP. This protein that is degraded by the MMPs generates the segregation of Aβ peptides, which are poorly processed. These peptides are considered to be mainly responsible for AD, generating the inflammatory component of the disease and, as a consequence, greater damage (induced by neuroinflammation) is generated at the cerebral level [48]. Astrocytes are CNS cells that are associated with the activity of neurons and neurotransmitters, causing synaptic transmission and neurovascular coupling [179]. MMP-2 and MMP-9 are expressed in astrocytes [180], and the expression of Aβ regulates the levels of MMPs secreted in the extracellular compartment, causing degradation [181]. The levels of MMP-2 and MMP-9 are significantly elevated in the presence of neuronal lesions [182]. The expression of MMP-2 and MMP-9 is stimulated by Aβ, and these proteins are secreted by astrocytes [183] and pro-inflammatory cytokines, such as IL-1β. [184]. MMP-2 is individually detected in CNS structures, such as the spinal cord, astroglia, and pyramidal neurons in the cortex and Purkinje cells in the external granular layer of the cerebellum. Increased expression of MMP-2 has been observed in astrocytes around the senile plaques of transgenic mice. This MMP is released in its latent form (pro-MMP-2), which requires activation by means of MT1-MMP, in contrast with MMP-9, which is expressed in the spinal cord, cerebellum, hippocampus, and cortex, as well as predominantly in the neurons [185]. Studies have reported a reduction in OS through the inhibition of MMPs, particularly in cases of cerebral amyloid angiopathy, as seen in AD [186]. Aβ 25-35 was shown to cause changes in the expression of MMP-9 and TIMP-1, and these changes were correlated with neurotoxicity [187]. Several studies have shown that patients with AD have a higher MMP-9:TIMP-1 ratio and a lower level of TIMP-1 in the cerebrospinal fluid (CSF) compared with cognitively healthy elderly individuals, and the MMP-9:TIMP-1 ratio in patients with AD correlates with T-tau in the CSF, a marker of neuronal degeneration [188]. The proliferative effect of Aβ 25-35 is increased by the presence of TIMP-1, suggesting that TIMP-1 is mainly secreted by injured neurons and plays a role in astroglial reactivity. Thus, stimulation of the MMP-9:TIMP-1 pathway by Aβ 25-35 fragments may represent a self-defensive mechanism of elimination of amyloid deposition from brains with AD [189]. MMPs and their inhibitors (TIMP-1 and TIMP-2) play roles in impaired amyloid-β peptide metabolism, which is responsible for the genesis and progression of neurodegenerative dementia [190]. TIMP-4 in plasma is associated with a significant risk of developing AD, demonstrating that TIMP-4 may reflect the severity of impaired cognitive function [191]. TIMP-3 can stabilize the Fas receptor by sensitizing it to FasL-induced apoptosis in AD [192]. TIMPs are located near the Aβ plaques and neurofibrillary tangles of brain samples affected by AD [193]. Similarly, it has been shown that MMPs are produced in excess at sites of injury by immune cells surrounded by the affected regions and that TIMPs can be located at these sites to control the activity of MMPs. However, the process by which this occurs is still not clear [48]. Thus, to validate the use of MMPs and TIMPs as possible candidates for the development of therapeutics, it is important to investigate whether they are amyloidogenic or prevent Aβ accumulation.

#### 4.4.4. Amyotrophic Lateral Sclerosis

Amyotrophic lateral sclerosis (ALS) is the most common progressive motor neuron disease and leads to death from respiratory paralysis [89]. The illness is progressive with a median survival time of 3 to 5 years [194]. The pathologic hallmark of motor neuron degenerative disorders is death of the lower motor neurons. A loss of corticospinal motor neurons is also observed. The motor neuron cytoskeleton is typically affected early in the illness [195]. Focal enlargements in proximal motor axons are frequent [196]. The death of the peripheral motor neurons in the spinal cord and brainstem leads to atrophy and denervation of the corresponding muscle fibers as well as the loss of the corticospinal tract [197]. A remarkable feature of the disease is the selectivity of neuronal cell death [198]. Pathological studies have revealed a proliferation of microglial cells and astrocytes in affected regions; in some cases, this phenomenon, known as neuroinflammation, can be visualized using positron emission tomography by scanning for ligands that are recognized by activated microglia [199]. In ALS, MMP-2, MMP-3, and MMP-9 can exert direct neurotoxic effects through the degradation of extracellular matrix proteins with consequent disruption of the BBB and promotion of inflammatory processes [200]. This results in the loss and damage of endothelial cells and astrocytes with all of these playing roles in the pathogenesis of motor neuron degeneration [201,202]. Increased levels of MMPs and TIMP-1 in patients with ALS may reflect the degeneration processes of motor neurons and skeletal muscles and/or are associated with tissue remodeling. Although the role of changes in the levels of MMPs and TIMPs in the pathogenesis of ALS is not clear, the analysis of these compounds in the serum may be used as a prognostic factor and potential marker for monitoring the effects of treatment [203]. A decrease in TIMP-2 in the stromal compartment of the bone marrow has been reported in patients with ALS [204]. TIMP-3 is considered an upstream mediator of neuronal apoptosis and likely contributes to neuronal loss in ALS patients [205].

### 4.5. MMPs and TIMPs in Cardiovascular Diseases

Cardiovascular diseases (CVDs) are considered to be the top causes of morbidity and mortality around the world [206]. MMPs are involved in the development and progression of atherosclerosis and other CVDs; therefore, their alteration is related to increased risks of cardiovascular morbidity and mortality [207]. In CVDs, alterations in the degradation and regeneration of the ECM occur due to instability of the vascular wall secondary to the damage seen in this type of disease [207]. MMPs have the ability to interact with different proteins intracellularly. MMP-2 is able to degrade a series of contractility-related myofilament proteins, causing a decrease in the Ca2+ sensitivity of myofilaments with subsequent contractile dysfunction, as seen in the scenario of ischemia–reperfusion [208]. The current literature surrounding the intracellular activities of MMPs focuses on their pathological roles, but little is known about their physiological functions in these contexts. After decades of study, we know that MMP-2, in particular, is abundant and actively retained within the cytoplasm [4,5,209,210,211,212,213], and MMP-2 participates in cardiac injury and repair [214,215,216]. It also can directly impair ventricular function in the absence of superimposed injury [216] and participates in uncharacterized physiological functions within the striated muscle, possibly in the maintenance of sarcomere proteostasis [4]. MMPs are considered important in cardiovascular diseases. MMPs are related to cardiovascular pathologies, such as aneurysm formation, coronary artery disease, myocardial infarction (MI), atherosclerosis, arterial hypertension, and heart failure (HF) [207,217,218,219]. TIMPs and MMPs play significant roles in tissue remodeling related to cardiac function [220]. TIMP-2 inhibits bFGF-induced endothelial cell proliferation, TIMP-3 inhibits cell proliferation and the migration of stimulated endothelial cells, and TIMP-4 inhibits endothelial cells. The use of inhibition by TIMPs as a therapeutic tool for vascular disease is under development [221].

#### 4.5.1. Hypertension

Clinically, according to the 2018 ESC/AHA guidelines for the management of arterial hypertension, hypertension is defined as a systolic blood pressure of more than 140 mmHg and a diastolic blood pressure of more than 90 mmHg. Epidemiologically, there is an overall prevalence of hypertension in adults of 30–45%. The presence of hypertension correlates with an increased risk of major cardiovascular events (hemorrhagic stroke, ischemic stroke, myocardial infarction, sudden death, heart failure, and peripheral artery disease) and end-stage renal disease [222]. The prevalence of hypertension is related to high dietary sodium intake, low dietary intakes of calcium and potassium [223], psychosocial stress, a low level of physical activity, and alcohol consumption [224]. In primary hypertension cases (95% of spontaneous cases), both non-innate (innate) and specific (adaptive) immune responses are activated. This leads to changes in the vascular walls in the microcirculation and then to chronic inflammation [129]. Inflammatory factors, such as C-reactive protein, IL-1β, IL-6, TNF-α, and ROS, are involved in the development and consolidation of hypertension by strengthening the stiffness of vessels and endothelial dysfunction [225]. MMP-2 and MMP-9 exhibit both pro- and anti-inflammatory properties. Elevated levels of MMP-9 and TIMP-1 in patients with essential hypertension are associated with increased arterial stiffness [226]. The activity of MMP-9 leads to the development of hypertension at an early stage, generating collagen degradation and arterial debilitation [227]. When an increase in blood pressure is generated, abnormal remodeling of the blood vessels occurs, and this later generates an overload of pressure in the cardiac muscle. MMP-9 plays a role in early-stage arterial remodeling in arteries, causing a rise in pressure through altered vessel distensibility. Under these conditions, vascular and cardiac tissue present additional compensatory remodeling, later leading to cardiac failure and other cardiovascular events [228]. As a result, the activity of MMPs increases during hypertension, which results in increased remodeling and progressive degradation of matrix components in the vascular wall; migration and proliferation of smooth muscle cells; and infiltration by monocytes [226]. Hypertension, as well as other inflammatory stimuli, has a double effect. On the one hand, it activates the T-lymphocytes, and on the other hand, it increases the production of chemokines and adhesion molecules in target tissues (heart, vascular, and renal), facilitating active access to pro-inflammatory cells [225]. Studies have shown elevated concentrations of TIMP-1 and MMP-9 in hypertensive subjects without HF and elevated levels of MMP-2 in hypertensive patients with HF [54]. TIMP-1 and MMP-1 are associated with hypertensive remodeling and correlate with extent of target organ damage (TOD) in patients with hypertension. MMP-9 activity can result in the increased degradation of elastin, leading to decreased elasticity, while decreased TIMP-1 activity can lead to the accumulation of unstable fibrin degradation products, which results in a misdirected deposition of collagen [229]. Some authors have suggested that increases in MMP-7 MMP-9, and TIMP-1 levels are predictive of the presence of left ventricular hypertrophy [49]. Investigations have examined hypertensive patients with left ventricular hypertrophy, observing higher levels of TIMP-2, TIMP-1, and TIMP-4. TIMP-1 seems to be correlated with the left ventricular mass and the degree of diastolic dysfunction [230]. In hypertensive subjects with paroxysmal atrial fibrillation, TIMP-1 is correlated with an increased left ventricular mass index, eccentric ventricular hypertrophy, and increased thickness of the interventricular septum. In hypertensive patients, TIMP-1 is associated with an unfavorable prognosis [231]. The decreased expression of TIMP-2 may be responsible for the abnormal deposition of ECM, structural remodeling, and atrial fibrosis [232]. A critical role for TIMP-3, among all TIMPs, is the preservation of the arterial ECM in response to Ang II. It is essential to recognize that Ang II-induced hypertension and TIMP-3 deficiency are not protective mechanisms, but they are due to adverse remodeling in the arterial matrix [233]. Dysregulated microRNAs are implicated in the progression of pulmonary artery hypertension. MiR-222 promotes pulmonary artery smooth muscle cell proliferation, at least partially, through targeting TIMP-3 [234]. The MMP-2:TIMP-4 ratio is detected as a marker of disease severity and right ventricular function, as well as a predictor of survival and time to clinical worsening; therefore, it could help guide the progression of the disease in patients with pulmonary arterial hypertension [40]. TIMP-4 can inhibit smooth muscle cell (SMC) migration and induce apoptosis in vitro and in vivo, which may generate new targets for the prevention and treatment of vascular diseases [74]. The results of several studies show the genetic diversity of MMPs and TIMPs as important factors that affect cardiovascular health and can be used to guide individual therapy. If MMPs play a significant role in these cardiovascular changes, the use of MMP inhibitors could be therapeutic in hypertensive patients at risk of developing cardiovascular complications.

#### 4.5.2. Atherosclerosis

Atherosclerosis is clinically defined as an inflammatory disease that involves the progressive accumulation of lipids and inflammatory cells within the intima of large arterial walls, leading to endothelial dysfunction [235]. The breaking of an atherosclerotic plaque is associated with the activity of MMP-9, which participates in the formation and destabilization of these atherosclerotic plaques that are formed by collagen types I, III, IV, V, XI, and XVI [236]. In atherosclerotic plaques, macrophages are the main source of MMP-9. The level of MMP-9 is also directly correlated with the activity of C-reactive protein, fibrinogen, and IL-6, which are factors that function as markers for predicting the risk of MI [237]. Fibrin is found in the glomerulus as a substrate for MMP-9, and it provides a protective effect through balancing fibrin degradation and preventing its accumulation in the glomerulus [238]. In addition, the inhibition of fibrin deposition by MMP-9 plays a role in the reduction of thrombus size and may be associated with a decrease in the progression of atherosclerosis [239]. TIMPs also seem to have an association with atherosclerosis. TIMP-4 is visible in cardiovascular tissue areas populated by inflammatory cells, mainly macrophages and CD3+ T-lymphocytes [240]. Human monocytes, macrophages, mast cells, and lymphocytes produce TIMP-4. In advanced atherosclerotic lesions, TIMP-4 is detected around necrotic lipid cores, and TIMP-3 is detected around and within the core regions, indicating that it has different roles in inflammation-induced apoptosis and ECM turnover. In cardiovascular disorders, TIMP-4 is associated with inflammation, suggesting its future use as a novel systemic marker for vascular inflammation. In atherosclerosis, TIMP-1 has been association with reductions in lesions [241]. Experimental studies have shown that a TIMP-1 deficiency is associated with macrophage-rich lesions with active proteinases and medial destruction. TIMP-2 inhibits the migration and apoptosis of macrophages and foam cells, as well as inhibiting atherosclerotic plaque development and destabilization, possibly through the modulation of macrophage and foam cell behaviors [242].

#### 4.5.3. Myocardial Infarction

Type 1 MIs are characterized by plaque disruption with coronary atherothrombosis, causing posterior myocyte necrosis [243]. The balance between MMPs and TIMPs is important, as alterations to the ECM composition can contribute to alterations to myocardial structure or geometry [220]. MMPs play a role in plaque stability, as they counteract the thickening of the intimal layer; however, if this becomes excessive, it can lead to obstruction and ischemia. MMPs also lead to the destruction of the major components of the ECM, which causes plaque rupture [244]. The MI environment is characterized by inflammatory cells (mainly neutrophils), inflammatory mediators, and MMP-9, which is related to the physiopathology of post-MI ventricular remodeling and congestive HF [245]. Research has found that neutrophils are a source of MMP-9 [246]. MMP-9 has been found to participate in post-MI ventricular remodeling through impaired angiogenesis and tissue remodeling [247]. Nonetheless, the deletion of MMP-9 stimulates neovascularization in remodeling myocardium rather than decreasing it [248]. Altered balance in the interactions of MMPs and TIMPs has been shown to play a significant role in the development of many diseases, including tissue remodeling after MI [220]. MMPs are upregulated following the development of HF post-MI. They can release ECM fragments called matricryptins that are key during the development of heart failure post-MI. Studies have suggested that matricryptins could be potential therapeutic targets for heart failure patients [249]. Due to the critical role played by MMPs during cardiac remodeling, the identification of the biological systems responsible for ECM synthesis and degradation within the myocardium holds great relevance in the progression of HF. TIMPs impact post-myocardial infarction remodeling [250] and correlate positively with the left ventricular mass and wall thickness [251]. TIMP-1 correlates with the echocardiographic parameters of left ventricular (LV) dysfunction after acute MI [252]. Studies have shown that after myocardial infarction, TIMP-3 improves remodeling and myocardial function, promoting angiogenesis and inhibiting early proteolysis. This demonstrates the therapeutic potential of preserving the local equilibrium of TIMP-3 in the heart given its diverse functions in the modulation of different processes involved in adverse post-myocardial infarction remodeling [71]. The plasma TIMP-4 concentration, measured early after MI, may assist in the prediction of LV remodeling and, therefore, in the assessment of the prognosis [253]. Other studies have shown higher levels of TIMP-1, TIMP-2, and TIMP-4 in the plasma after AMI and are associated with major adverse cardiac events (MACEs) [65]. TIMP-2 deficiency accelerates adverse post-myocardial infarction remodeling because there is enhanced MT1-MMP activity, despite a lack of MMP-2 activation [250]. On the other hand, studies have shown that miR-17-3p increases the proliferation of cardiomyocytes and cell size by targeting TIMP-3 and acting upstream of the PTEN-AKT pathway. MiR-17-3p may represent a new therapeutic target to promote functional recovery after cardiac ischemia–reperfusion [254].

The imbalance between TIMPs and MMPs has been recognized as a factor that contributes to post-MI remodeling, so therapeutic strategies targeting this imbalance are necessary.

#### 4.5.4. Aortic Aneurysms

An aneurysm is defined as an abnormal blood-filled dilation of the blood vessel wall, resulting from disease of the vessel wall. Some of its complications are rupture, stenosis, thrombosis, and embolism [255]. Aortic aneurysms result from conditions that cause abnormal production of the structural components of the aortic wall: collagen and elastin. Oxidative stress, inflammation, biomechanical wall stress, and proteolysis contribute to the degenerative processes that characterize the development of most aneurysms [256]. These are mediated by macrophages, T- and B-lymphocytes, inflammatory cytokines, and MMPs that degrade collagen and elastin, altering the tensile strength and ability of the aorta to accommodate pulsatile stretch. Factors associated with the occurrence of degenerative aortic aneurysms include aging, cigarette smoking, hypercholesterolemia, hypertension, and male sex [257]. The most common pathological condition associated with degenerative aortic aneurysms is atherosclerosis. Many patients with aortic aneurysms have coexisting risk factors for atherosclerosis, as well as atherosclerosis in other blood vessels [258]. Familial clustering of aortic aneurysms occurs in 20% of patients, suggesting a hereditary basis for the disease. Mutations of the gene that encode fibrillin-1 are present in patients with Marfan’s syndrome [259].

Physiologically, an aneurysm wall has decreased elastin and increased collagen in its structure, due to the degradation of elastin, mainly by MMP-2 and MMP-9 [260,261]. MMP-2 and MMP-9 also have the ability to degrade denatured fibrillary collagen and collagen types IV, V, and VII [262]. Infiltrates of inflammatory cells have been reported in different stages of aortic aneurysm formation and involve immune reactions in the aneurysm wall [263] primarily involving macrophages, T-lymphocytes, and pro-inflammatory cytokines [264]. This gradually induces aortic dilation, leading to progressive destruction of the normal laminar architecture and fragmentation of elastin fibers [265].

MMP-2 is synthesized by smooth muscle cells and has been reported as a primary factor in aneurysm etiology [266]. MMP-9 is synthesized by inflammatory cells, such as macrophages and neutrophils [267]. MMP-12, synthesized by macrophages, has been implicated in the degradation of elastin [268]. Elevated levels of MMP-9 and MMP-2 are responsible for the formation and size of aneurysms, as they contribute to the degradation of elastin [269]. Differential expression, with MMP-9 increased and both TIMP-1 and TIMP-2 reduced, occurs in the most common forms of thoracic aortic aneurysms [270]. Local expression of TIMP-1 prevents aneurysm progression and rupture [271]. Greater expression of TIMP-4 in aneurysms has been shown. TIMP-4 counteracts MMP-2, allowing a balanced matrix turnover to be achieved. The difference in the MMP-2:TIMP-4 ratio reflects this balance in patients with aortic aneurysms [272]. Low levels of both TIMP-2 and TIMP-1 in aortic aneurysms may represent a favorable environment for aneurysm expansion and collagen degradation [273].

### 4.6. MMPs and TIMPs in Gynecological Disorders

MMPs, cytokines, and growth factors intervene in physiological processes such as embryo implantation, trophoblast invasion and migration, and decidualization, with studies referring to blastocysts as being a direct source of MMP-2 [206]. Among the cytokines involved in decidualization, IL-11, which is produced by stromal and epithelial cells and increased by IL-1α, TNF-α, and TGF-β, induces the production of the human endometrium [274]. In addition to the involved cytokines, elevated levels of MMPs, such as MMP-2, MMP-3, and MMP-9, expressed in the decidua are associated with an increase in trophoblast invasion [41]. The presence of MMPs, along with gonadotropin chorionic hormone and VEGF, allows for modifications to the endometrium that are required for human implantation to occur, as well as playing a role in the prevention of abnormal pevelopment of the placenta [41,275].

MMPs and TIMPs control the site and remodeling of ovarian tissue and are associated with a variety of physiological and pathological processes. The correct balance between MMPs and their inhibitors plays a large role in the structural and functional vascular changes of women with complicated pregnancies [276].

#### 4.6.1. Polycystic Ovarian Syndrome

Polycystic ovarian syndrome (PCOS) is the most common disorder of endocrine origin in women of reproductive age. Its clinical expression commonly includes oligo/anovulation, hyperandrogenism (clinical or biochemical), and the presence of polycystic ovaries, and it has a stretch relationship to insulin resistance, diabetes mellitus type 2, obesity, cardiovascular disease, and cancer risk [277]. PCOS is one of the most common causes of anovulatory infertility and affects 6–10% of premenopausal women. For diagnosis, adrenal and pituitary disorders must be excluded, and the counting of primordial follicles is also important [278]. PCOS has an important incidence in young women, and it can be underdiagnosed due to greater levels of follicles. Its etiology is not clear, but there is evidence of a genetic factor that affects patients during oogenesis [279]. The increasing levels of androgens that are seen in PCOS patients lead to insulin resistance in these patients, activating the synthesis of androgens and LH production, suppressing the synthesis of the sex hormone binding globulin, and stimulating adrenal and androgen biosynthesis related to anovulatory cycles [280]. Studies have investigated MMP-2 and MMP-9 expression in the ovaries [281]. Elevated concentrations of MMP-9 have been reported in women with PCOS that are related to the occurrence of angiogenesis and endothelial dysfunction seen in PCOS, as well as normal functions such as follicular growth, ovulation, and establishment of the luteal corpus [282]. One of the histological characteristics of PCOS is follicular atresia and the formation of ovarian cysts. MMP-9 is related to an imbalance in the excessive reconstruction of the ovarian follicles and walls, causing irregular ovulation, follicular occlusion, and an increase in stromal tissue [283]. TIMP-1, TIMP-2, and TIMP-3 are located in the stroma and teak of developing follicles. In addition to serum concentrations of MMP-9, MMP-9:TIMP-1 ratios are significantly higher in women with PCOS than in healthy women [284]. An altered balance between serum levels of MMPs and TIMPs in women with PCOS has recently been reported. Namely, there is a decrease in the serum level of TIMP-2, and the proportions of MMP-9 to TIMP-1 and MMP-2 to TIMP-2 increase significantly [68]. TIMP-1 levels may especially reflect both systemic chronic inflammatory and immune responses. Increased levels of MMP-8 and the MMP-8:TIMP-1 ratio in saliva and serum seem to be more pronounced in women with PCOS, and they are potentiated by gingival inflammation [285]. In addition, studies have shown that testosterone levels correlate positively with the proportion of MMP-9 to TIMP-1 and they correlate negatively with TIMP-2 [286]. It can be assumed that excessive androgen secretion can alter the balance of MMPs and TIMPs in the ovaries under physiological conditions, resulting in the progression of fibrosis in patients with PCOS [287]. Repression of the MMP-9:TIMP-1 ratio may have an important modulatory effect on progesterone secretion [288]. On the other hand, other studies have shown that the concentration of TIMP-3 mRNA is significantly lower in polycystic ovaries. The alterations observed in the production and/or distribution of type IV collagen and TIMP-3 suggest the involvement of the basement membranes in the pathogenesis of PCOS [72]. High levels of TIMP-1 have been seen in PCOS patients, and women who achieve pregnancy have higher TIMP-1 levels [66].

#### 4.6.2. Spontaneous Abortion

Spontaneous abortion (SA) is defined as the expulsion of the fetal products before they become viable at a gestational age of at least 22 weeks or when the fetus achieves a weight of 500 g (mostly between the 6th and 10th weeks of pregnancy) [289]. SAs are most common in women aged between 20 and 45 years, with their climax occurring at the ages of 40 to 44 years (90%) [290].

The main causes of SA are maternal, ovofetal, and implantation factors [291]. Regarding implantation, the early loss of a fetus increases when implantation takes longer than 10 days, but another 20% of cases occur when the trophoblast fails to implant. The most common problems occurring between 0 and 10 weeks gestation relate to fertilization or malformations in the fetus caused by chromosomal abnormalities [292]. There are conditions that halt the correct development of the fetus, such as systemic maternal disease, infections, congenital defects in the reproductive system, or psychosomatic affections [293]. Clinical manifestations of SA include hemorrhage in the decidua caused by implantation failure, resulting in the expulsion of the products (between 14 and 22 weeks gestation). Retention in the cavity may also occur before 8–14 weeks of gestation or a rupture of membranes with the expulsion of the fetus but not the placenta, which remains in the uterine wall and causes significant hemorrhaging [278].

Human pregnancy requires hemostasis to prevent hemorrhage, as wall as normal placentation, decidualization, and modification of the spiral arteries. The upregulation of MMPs and cytokines are directly related to spontaneous preterm birth [294]. MMP and TIMP signaling and their pleiotropic functions are required for a normal pregnancy [295]. The expression of MMPs and TIMPs during trophoblast invasion, directed by trophoblastic cells, is required. MMP-9, expressed by trophoblast cells in the embryo, and TIMP-3, expressed by maternal uterine cells, are the main enzymes involved in the invasion of trophoblast cells in the endometrium, being essential participants in the implantation process and correct development of embryos [296,297]. The synthesis of MMP-2 begins at the beginning of gestation, with its main function being the invasion of the trophoblastic bed [298]. The upregulation of MMP-9, MMP-2, and TIMP-2 plays a role in early spontaneous termination of pregnancy, usually before 12 weeks of gestation [299]. A study by Anumba et al. found elevated levels of TIMP-2 in women with a history of recurrent pregnancy loss (RPL), linking it to abnormal implantation and development of the placenta, as well as being a predictive marker for RPL [300]. The expression levels of MMP-9 mRNA and the MMP-9:TIMP-3 mRNA ratio are also associated with SA [297]. Dysregulated TIMP-1 expression is associated with infertility and early pregnancy loss [301]. Studies have shown that decidual stromal cells expressing CD82 upregulate the expression of TIMP-1 in an autocrine manner and inhibit the invasiveness of first-trimester human trophoblast cells, partly through the β1 integrin/MAPK signaling pathway [302].

#### 4.6.3. Preeclampsia

Preeclampsia (PE), as part of hypertensive disorders in pregnancy, is considered a serious complication worldwide. It is characterized by new-onset hypertension in the second half of pregnancy accompanied by proteinuria or thrombocytopenia, renal insufficiency, impaired liver function, pulmonary edema, and cerebral or visual symptoms. These symptoms are due to poor placentation that results in vascular dysfunction and major cardiovascular complications [303]. MMPs are associated with both implantation of the embryo and invasion of the trophoblast into the endometrium and inner myometrium. Therefore, a deregulation of MMP activity is related to abnormal placentation and vascular dysfunction, as seen in PE [304]. During the first weeks of pregnancy, MMPs begin preparing an adequate environment in the placental bed. The concentrations of MMP-2 and MMP-9 are maintained throughout pregnancy with the main function being to intervene during implantation and carry out trophoblastic invasion (MMP-9) and maintenance of the placental bed (MMP-2) [305]. The invasion of trophoblastic cells is led by autocrine and paracrine stimuli from the uterine and trophoblastic cells. In addition, several molecules that have been implicated in PE development, such as proteinases, cytokines, and growth factors, play roles in the excessively shallow invasion of the trophoblast seen in the physiopathology of PE, causing disturbed and inadequate remodeling of spiral arteries and thus reducing blood flow to the intervillous space, leading to systemic hypertension and fetal hypoxia [306]. In a study by Plaks et al., a mouse model of PE was reproduced by inactivating maternal and embryonic MMP-9, demonstrating a requirement for properly balanced MMP-9 activity in normal placentation [307]. Decreased MMP-2 and MMP-9 expression/activity leads to decreased vasodilation, increased vasoconstriction, and inadequate remodeling of uterine spiral arteries, resulting in impaired perfusion of the fetus–placental unit, leading to the secretion of factors within the maternal circulation and the establishment of endothelial dysfunction [308]. Studies have found increased MMP-2 activity/levels in patients with PE, as it can promote platelet aggregation, vascular remodeling, and the release of vasoactive peptides, such as entothelin-1. These concentrations have been shown to be mediated by vascular endothelial growth factor (VEGF) [309]. MMP-1 is elevated in women with PE, but without an increase in TIMP-1. It is involved in the degradation of vascular collagen type I, which increases vascular permeability and favors edema and proteinuria, which are features observed in patients with PE. There is a stretch relationship between the neutrophil activation, migration, and vascular dysfunction seen in PE, as MMP-1 has roles in leukocyte migration and vascular dysfunction; likewise, its expression is induced by activated neutrophils, which also generate ROS and TNF-α [310]. The levels of MMPs in the urine, particularly MMP-2, have been postulated as predictive biomarkers for patients at high risk for PE between 12 and 16 weeks of gestation. This shows that an existing imbalance of MMPs in pregnancy can affect the vasculature at structural and functional levels, with detectable changes occurring before the disease and its clinical signs appear [311]. The controlled expression and proper balance between MMPs and TIMPs, which determine the net MMP activity and hence regulate the invasive potential of placental trophoblasts, are pivotal for maintaining normal placentation [312]. In general, any imbalance in this equilibrium can lead to the development of pathological conditions, and specifically, during pregnancy, this imbalance disturbs the trophoblastic invasive cascade [298]. TIMP-1 is a major endogenous inhibitor of MMP-9 that may affect the responsiveness of hypertensive disorders of pregnancy to therapy [313]. TIMP-1 is elevated in PE [314]. Studies in placentas from pregnant women diagnosed with PE have shown that at the transcriptional level, the abnormal expression of TIMP-1 can cause insufficient trophoblastic invasion and superficial placentation [315]; However, other studies have shown reduced MMP-1 levels in the umbilical serum, placenta, and decidua in women who have developed PE [316]. TIMP-2 levels are higher in women who develop a hypertensive disorder [317]; however, the specific role of TIMP-2 in the disease is still unknown. TIMP-3 expression is markedly reduced in preeclamptic placentas, as TIMP-3 is an endogenous TACE inhibitor, and the downregulation of TIMP-3 activity in trophoblasts could result in increased TACE expression and subsequently lead to increased TNF-α production [318]. Histone deacetylases promote trophoblast cell migration and invasion by repressing the TIMP-3 promoter through histone hypoacetylation [319]. Furthermore, studies have shown that the TIMP-3 promoter is hypomethylated in the placentas of women with PE, suggesting that epigenetic alterations may be associated with reduced trophoblastic invasion [320]. Hypomethylated and placenta-specific TIMP-3 may be a potential marker for the early diagnosis of PE in maternal plasma. In PE, there are elevated levels of circulating TIMP-4 compared with those in healthy pregnant women, and these levels are correlated with clinical parameters of disease. Other studies have shown that plasma TIMP-4 levels are not altered before the manifestation of clinical symptoms; therefore, they are not good predictors of the development of PE [321]. It has been suggested that polymorphisms in TIMP-4 protect against the development of PE [322] and that TIMP-4 is a downstream target of the transcription factor glial cell missing-1 (GCM1) that may link the consequences of reduced GCM1-directed trophoblast differentiation to histological and functional components of disordered placentation in severe PE cases [323].

### 4.7. MMPs and TIMPs in Cancer

Cancer constitutes an enormous burden on society, as it is a major cause of mortality and morbidity worldwide [324]. MMPs and TIMPs play important roles in multiple stages of tumorigenesis. A disruption in the balance between MMPs and TIMPs has been implicated in the progression of cancer [325].

Initially, in cancer biology, MMPs were thought to participate in the beginning of metastasis, due to the disintegration of the physical barriers that correspond to the ECM and basement membrane collagen. However, now it is known that MMPs play roles in all steps of tumor progression, influencing multiple biological functions, including the modification of signaling pathways, the regulation of cytokines involved in the immune response, and tumor growth (particularly by generating angiogenesis and therefore the spread of cancer) [326]. The activity of MMPs in the tumor microenvironment increases due to their roles in the remodeling of the ECM, the regulation of biological activity, and the availability of cytokines and growth factors. Depending on the MMP involved and the tissue site where a tumor is located, they can also have tumor-suppressive effects; an example of this is provided by MMP-8 and MMP-12, which are recognized as anti-tumor MMPs [327]. MMP-11 exerts a dual effect on tumors. MMP-11 promotes cancer development by inhibiting apoptosis and enhancing the invasion of cancer cells. On the other hand, in animal models, MMP-11 plays a negative role against cancer development via the suppression of metastasis [328]. It is important to highlight this dual effect because MMP-11 could function as a significant tumor biomarker in cancer for tumor staging, prognostic analysis, monitoring of recurrence during follow-up, for immunotherapy, and for early detection.

The process for pre-metastatic development at a predetermined location is dictated by the remodeling of the ECM. MMPs are involved in the release of soluble factors involved in additional cellular recruitment as well as in the expression of growth factor receptors in metastatic cells. These growth factors include vascular endothelial growth factor receptor 1 (VEGFR1) in bone marrow-derived hematopoietic progenitor cells. MMP-9 has been found to be overexpressed by endothelial cells and myeloid cells [329]. Tumor cell metastasis is facilitated by the pre-metastatic niches represented by bone marrow-derived cells (BMDCs) and type IV collagen in the basement membrane. This type of collagen is released by a primary tumor in response to the hypoxic conditions in the tumor environment, where BMDCs degrade collagen type IV through the expression of MMP-2, which contributes to invasion and metastatic growth [330].

There have been large investments by pharmaceutical companies for the development of MMP inhibitors to treat cancer. However, many of them have flopped, indicating that there is still so much to elucidate regarding the association of MMPs and cancer. In spite of the negative factors, experts in the field are still working to develop useful anti-MMP drugs [331] (see more information in the “Targeting MMP and TIMP function (inhibitors)” section).

The TIMP family inhibits the proteolytic activity of several MMPs involved centrally in the invasion of tumors and metastases; however, TIMPs can also participate in tumor growth, apoptosis, and angiogenesis [332]. All TIMPs are secreted, but only TIMP-3 is incorporated into the matrix. The glycosylation of TIMP-3 increases its affinity for glycan-bound MMPs. Aberrant glycosylation is widespread in cancer [333]. Although TIMP-3 antagonizes matrix metalloproteinase activity and can suppress tumor growth, angiogenesis, invasion, and metastases [334], TIMP-3 also has other biocellular functions not related to MMP inhibition, such as the induction of apoptosis [335,336] and the inhibition of endothelial cell motility and proliferation [337]. In contrast to TIMP-1 upregulation, there is overwhelming evidence for the occurrence of TIMP-3 silencing in multiple human cancers [338,339,340,341,342].

Several authors have shown that silencing of the *TIMP-3* gene is associated with an aberrant methylation of the promoter region and is correlated with the loss of TIMP-3 expression [334]. Low expression of TIMP-3 leads to the increased activity of MMPs in cancer [335]. Several studies have shown that that high levels of TIMP-3 mRNA are associated with a good prognosis in different types of cancer [339,341,342,343,344,345,346]. In addition to its MMP inhibitory function, it is also known that TIMP-1 promotes cell growth and proliferation and inhibits apoptosis, and it is probably involved in the regulation of angiogenesis. Elevated levels of TIMP-1 mRNA and TIMP-1 protein are found in different types of cancer, and several clinical studies have shown positive associations of high TIMP-1 expression with a poor prognosis or tumor progression in lung, brain, prostate, breast, colon, and other cancers [61,67,347,348,349,350,351,352]. The role of TIMP-1 in cancer is controversial, because it can have both pro- and antitumoral effects. Compared with the constant and opposing patterns of TIMP-1 and TIMP-3, variable trends have been reported for TIMP-2 and TIMP-4 in human cancer, which could due to the heterogeneity of the types and stages of cancer [8]. TIMP-2 diminishes cell proliferation, neoangiogenesis, and tumor growth. Both the overexpression and reduction of TIMP-2 have been documented in several clinical studies of breast, lung, gastric, and colorectal cancers. TIMP-2 can also be epigenetically silenced by promoter hypermethylation in prostate cancer [353]. TIMP-2 expression indicates a favorable prognosis in some types of cancer [354,355,356,357]. On the contrary, there are studies showing an association of higher TIMP-2 levels with a poor prognosis [358,359,360]. Similar to TIMP-2, it has been found that TIMP-4 is upregulated or downregulated in several types of cancer. Studies conducted in breast and prostate cancers have shown an increase in TIMP-4 in the early stages of disease but a decrease at the end in the invasive stages. In relation to other TIMPs, there have been fewer reports of changes in TIMP-4 [63,75,76,77].

TIMP deregulation causes an imbalance in crucial signals that initiate pathways contributing to the cancer hallmarks of sustained proliferative signaling, the evasion of growth suppressors, and cell death resistance, enabling replicative immortality [8].

At present, specific alterations that are known to be involved in the progression of cancer, such as (a) proliferation, (b) survival, (c) angiogenesis, (d) enabling replicative immortality, (e) invasion/migration, and (f) immunity evasion [331,361], demonstrate that MMPs and TIMPs are involved in different processes of carcinogenesis and not only in the processes of invasion and metastasis [6].

(a) Cell growth regulation. MMP-1, MMP-2, MMP-3, and MMP-7 are able to control the growth of tumor cells by mechanisms such as the release of precursors of growth factors that are attached to the cell membrane, modulating the bioavailability of growth factors and regulating cell proliferation signals [362]. MMPs inhibit tumor growth through the activation of TGF-β and through the generation of proapototic molecules [6]. TIMPs are believed to possess several cellular functions, particularly the contrasting activities of inhibiting tissue-degrading enzymes and promoting cellular growth [355]. TIMP-1 and TIMP-2 show cell growth-promoting activity in various cell types, including keratinocytes and fibroblasts [363]. In an investigation of cell events associated with TIMP-induced cell growth, it was found that both TIMP-1 and TIMP-2 increase the level of Ras-GTP, but they use different signaling pathways. TIMP-1 activates the tyrosine kinase/mitogen-activated protein kinase signaling pathway, while TIMP-2 signaling is mediated by the activation of protein kinase A, which is directly involved in the formation of the Ras/phosphoinositide 3-kinase (PI3-K) complex [364,365]. Some studies have suggested that the effect of TIMP-2 on cell growth is mediated through its binding to MMP-14 on the cell surface and the subsequent activation of extracellular-regulated kinase (ERK) 1/2 [366]. However, TIMP-2 has distinct functions and properties that are independent of its protease inhibitory activity. These properties include the activation of Shp-1 protein tyrosine phosphatase activity and inhibition of tyrosine kinase receptor signaling by binding to α3β1 integrin on the cell surface. This mechanism of receptor inactivation results in the inhibition of growth factor-mediated proliferation of both tumor (fibroblasts and endothelial cells) and normal cells [354]. TIMP-2 also inhibits growth by activating adenylate cyclase, increasing intracellular cyclic adenosine monophosphate (cAMP) levels, and activating the SH2-tyrosine phosphatase-1 (SHP-1) protein, which reduces the activity of growth factor receptors and inhibits the activation of MAPK [367].

(b) Regulation of apoptosis. MMP-7 and ADAM10 confer antiapoptotic activity to cancer cells by cleaving the Fas ligand from the cell surface [368]. The antiapoptotic activity of the MMPs is also influenced by proteolytic detachments of proteins related to the tumor-associated major histocompatibility complex (MHC) class I [369]. TIMP-1 is an inhibitor of apoptosis [364], and it binds to the CD63 receptor and interacts with the β1 subunit of the integrins [370]. Consequently, the TIMP-1/CD63/β1 integrin complex constitutively activates the survival signals through the activation of focal adhesion kinases (FAKs), PI3-K, and ERK [371,372]. TIMP-3 binds to the ECM after secretion, allowing it to control pericellular proteolysis through its potent inhibition of MMP, ADAM, and ADAMTS [62]. Some studies have proposed that the proapoptotic effects of TIMP-3 result from the inhibition of TACE (ADAM17) with the subsequent stabilization of TNF receptors [373]. Other investigations have confirmed that TIMP-3 stabilizes three distinct death receptors: TNF receptor 1, FAS, and TNF-related apoptosis inducing ligand receptor 1 (TRAIL-R1) [374].

(c) Regulation of angiogenesis. Angiogenesis is essential for tumor propagation. MMPs, mainly MMP-2, MMP-3, MMP-10, MMP-11, MMP-1, MMP-8, and MMP-13, have two functions in this process, initially promoting (but at the same time, they have a role in inhibiting) angiogenesis [375]. MMPs initiate the process of angiogenesis through the degradation of type IV collagen in the basement membrane, in particular, MMP-9, which relseases angiogenic factors, such as VEGF and fibroblast growth factor-basic (FGF-b), that are bound to the ECM [376]. Conversely, MMPs also inhibit the process of angiogenesis by promoting the cleavage of type XVIII collagen, producing endotastine and releasing urokinase-type plasminogen activator receptors that are bound to the cell surface, which are required for the invasion from endothelial cells to fibrin [377]. It is now well-established that all four TIMPs have antiangiogenic activity [378]. TIMP-1 is probably involved in the regulation of angiogenesis, although this remains controversial [379]. The antiangiogenic activity of TIMP-2 is mediated through its interaction with α3β1 integrin. Studies have demonstrated that the overexpression of TIMP-2 inhibits tumor growth and angiogenesis by the upregulation of MAP kinase phosphatase 1, which dephosphorylates p38 MAPK, a molecule involved in the proliferation and migration of endothelial cells [380]. In addition, TIMP-2 improves the expression of RECK, a membrane-anchored protein with inhibitory activity for MMP-2, MMP-9, MMP-14, and ADAM10 [381]. The antiangiogenic activity of TIMP-2 has been localized to a region of a sequence in the C-terminal domain [382], which may be the site of interaction with α3β1 integrin. TIMP-3 is also a potent inhibitor of angiogenesis. It binds directly to VEGF receptor 2 and blocks the action of VEGF on endothelial cells [383]. TIMP-3 also binds to angiotensin II type 2 receptor, and the overexpression of both TIMP-3 and angiotensin II type 2 receptor additively inhibits angiogenesis [384]. There is some evidence that interactions between endothelial cells and pericytes stimulate the expression of TIMP-3 in pericytes. TIMP-3 from this source, along with TIMP-2 expressed in endothelial cells, participates in vascular stabilization by inhibiting several MMPs and ADAMs [385] and, presumably, by interacting with VEGFR-2 [383].

(d) Regulation of invasion and metastasis. Tumor propagation occurs through multiple events, where cell motility and migration combine with proteolysis, tumor cell proliferation at a new site, and angiogenesis, and it is associated with the interaction of cells with the ECM [386]. MMPs play a role in clearing pathways for tumor cell invasion and metastasis, as well as tumor growth, by providing oxygen and nutrients for the tumor through disruption of the basement membrane and ECM [387]. MMPs are involved in cell invasion and migration with the later establishment of colonies in predicted distant tissue sites. Mainly with the help of MMP-2 and MMP-9, they manage to degrade the type IV collagen of the basement membrane and thus gain access to the blood and lymphatic vessels [388].

The overexpression of each TIMP in cancer cell lines inhibits their migration, invasion, metastasis, and subsequent growth [8]. Several MMPs can cleave cell adhesion proteins, such as cadherins, which mediate cell–cell contact and integrins [389]. TIMP-1 is an inhibitor of ADAM10, and TIMP-3 is the only inhibitor of ADAM17. Their overexpression inhibits NOTCH signaling [80]. Transcription factors such as TWIST1 and SNAIL create a shift in cadherin expression (that is, E-cadherin loss or N-cadherin gain) that facilitates motility and cell depolarization. SNAIL leads to the induction of TIMP-3, and similarly, although independent of the metalloproteinase inhibitory function, the expression of TIMP-1 induces TWIST1 to negatively regulate E-cadherin in cancer cells [390], while TIMP2 upregulates this cadherin. In fibroblasts, the absence of TIMP1 [391] and TIMP3 [392] compromises the function of cadherin and the cell–cell contact. Similarly, the absence of TIMP-3 causes aberrant cellular distribution of E-cadherin and related β-catenin signaling [392]. There is evidence to show that the release of TIMP-1, TIMP-3, or TIMP-4 inhibits the invasive process, while the release of TIMP-2 increases this process [392]. Although the TIMP−metalloproteinase balance can control the matrix alignment, density, and rigidity, the evidence supporting this concept is quite limited. Lysyl oxidase (LOXL) crosslinking of collagen fibrils promotes tumour cell motility [221]. The elimination of LOXL2 in cancer cells decreases its invasion and TIMP-1 levels. It is believed that the development of a metastatic niche is based on specialized components of the ECM that include tenascin and periostin proteins [393], and the overexpression of the isoforms of tenascin alters the expression of TIMP-3 and MMPs [394].

**Figure 2:**
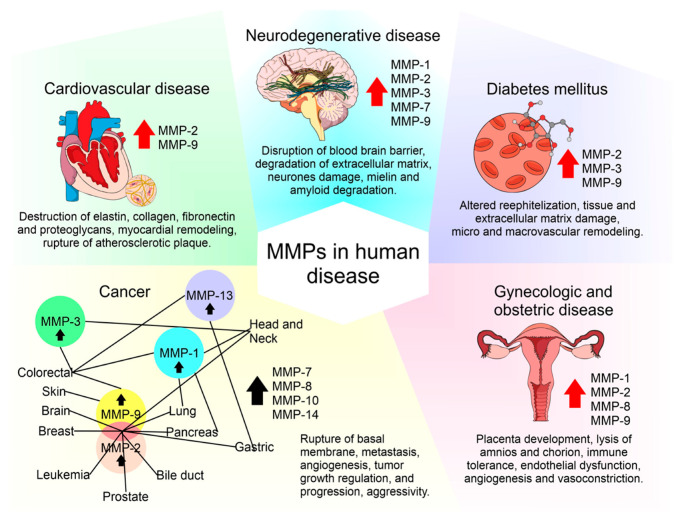
MMPs play distinguishing roles in the pathogenesis of multiple common human diseases. MMP-2 and MMP-9 are the most common MMPs related to several diseases. In cardiovascular diseases, they have been implicated in the pathogenesis of aortic aneurysms, the instability of atheroma plaques with posterior rupture and acute myocardial infarction, and later myocardial remodeling. In neurodegenerative diseases, they induce a disruption in the blood−brain barrier (BBB) with consequent neurotoxicity and degradation of the extracellular matrix (ECM), causing global neuron damage. In diabetes mellitus, they alter re-epithelization in wounds, and they have roles in the development of diabetic nephropathy through glomerular hypertrophy and posterior glomerulosclerosis. MMPs have been implicated in every step of cancer progression, including tumor growth regulation, proliferation, and invasion/metastasis through MMP-2 and MMP-9, the creation of a pre-metastatic niche, angiogenesis, and other activities in the tumor microenvironment, such as the chemotaxis of inflammatory cells and the propagation of the inflammatory microenvironment. In obstetrics and gynecology, they intervene in normal and pathological contexts, such as the delivery and spontaneous rupture of fetal membranes as well as in the trophoblastic invasion phase during embryo implantation, and their altered activity has been implicated in the development of diseases such as preeclampsia and polycystic ovarian syndrome. Some elements of this figure were taken from the Mind the Graph platform, available at www.mindthegraph.com. Red and black arrows mean overexpressed.

**Figure 3:**
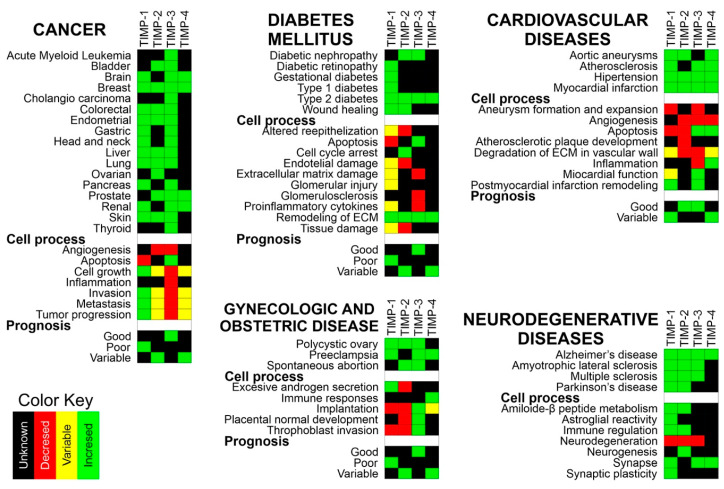
Role of TIMPs in the pathogenesis of multiple common human diseases. An increase in TIMPs and their expression causes different effects within specific tissues. TIMP-1 and TIMP-3 are the most common TIMPs found in different human diseases. In cancer, diabetes mellitus, and gynecological diseases, TIMP-1 is considered to be a marker of poor prognosis, because it is associated with disease progression. The main cellular processes that involve TIMP-1 are the promotion of cell growth and antiapoptotic activity. In the same diseases, TIMP-3 is considered to be a marker of good prognosis, because it restricts disease progression. The cellular processes in which it participates are the reduction of angiogenesis, the suppression of cell growth and inflammation, and the increase of apoptosis. TIMP-3 may even suppress the earliest aspects of disease development. In neurodegenerative and cardiovascular diseases, the roles of TIMP-1 and TIMP-3 are variable. TIMP-2 and TIMP-4 are variable prognostic markers in different diseases. In diabetes mellitus, TIMP-2 and TIMP-4 promote the remodeling of the ECM. In neurodegenerative diseases, TIMP-2 promotes cell cycle arrest and immune regulation. In cardiovascular disease, TIMP-4 promotes apoptosis and inflammation. Within gynecological diseases, TIMP-4 promotes the immune response. Furthermore, TIMP-2 decreases implantation, normal placental development, and trophoblastic invasion, while TIMP-3 promotes these cellular processes.

(e) Regulation of immune surveillance. MMPs play a role in immunity evasion, and they activate TGF-β, indirectly managing the function of T-lymphocytes. This means that in cancer tumors, MMPs can indirectly affect the action of leukocytes, because chemokines are direct targets of MMPs that prevent these molecules from acting as chemotactic attractants [395]. The evolution of a tumor incorporates abnormal cell proliferation and death signals together with the inflammation that promotes the tumor, and the pleurotropic cytokine TNF-β has the ability to initiate these three processes. Similar to the physiological inhibitor of ADAM17, TIMP-3 not only regulates TNF binding to the membrane against systemic TNF but also its two receptors (TNFR1 and TNFR2), which are released into the blood. It has been shown that the absence of TIMP-3 causes an increase in TNF signaling and the inflammatory response as a result of the deregulated activity of ADAM17 [80]. TIMP-3 is essential for limiting inflammation. A loss of TIMP-3 may increase IL-6 production via the tumor necrosis factor-α/nuclear factor κB axis [73].

(f) Regulation of inhibitory growth signals. Determination of the roles of MMPs and cancer progression through proteolysis has generated the development of drug inhibitors of these enzymes [396]. Initially, the transcription of MMPs is inhibited, continuing to block the pathway that directs the transduction that generates signals, such as the MAPK pathway and ERK pathway, thus sending nuclear factors to inhibit the expression of MMPs [396]. TIMPs can be regulated at the transcriptional level by various cytokines and growth factors, resulting in tissue-specific, constitutive, or inducible expression. Expression of the TIMP-1-encoding gene is regulated by cytokines, including IL-1, TNF-α, TGF-β, IL-6, and IL-10. The expression of TIMP-3 is regulated by TGF-β. Other inhibitors (TIMP-2 and TIMP-4) are less susceptible to the effects of cytokines [225]. The overexpression of specific miRs strongly potentiates the initiation and progression of cancer by suppressing TIMP proteins. miR-221 and/or miR-222 can attack TIMP-3, inducing drug resistance and facilitating cell growth in different types of cancer through ADAM10- and ADAM17-dependent signaling [8]. Members of the miR-181 family suppress TIMP-3. Interestingly, it has been shown that TGF-β, whose activity is regulated by TIMPs, induces miR-181 through SMAD4 [8]. The miRs involved in inflammatory diseases, such as miR-21, also participate in the link between inflammation and cancer [8]. Several studies have shown that the expression of miR-21 induces the silencing of TIMP-3 [397]. miR-196 suppresses TIMP-1 and increases the expression of MMP-1 and MMP-9 indirectly through the mitochondrial protein nucleoside diphosphate kinase 4 (NME4) and N-terminal kinase of June [80]. The miRNA−TIMP axis has been proposed as a therapeutic target for aggressive or drug-resistant human cancers. Therefore, new knowledge about the participation of MMPs and TIMPs in all stages of cancer progression and the tumor microenvironment could open up new, more directed strategies for cancer treatment as adjuvant therapies in early or advanced stages of disease [398]. Figure 2 and Figure 3 illustrate the participation of MMPs and TIMPs in the different human diseases previously mentioned.

### 4.8. MMPs and TIMPs in Inflammatory Diseases

**Figure 4:**
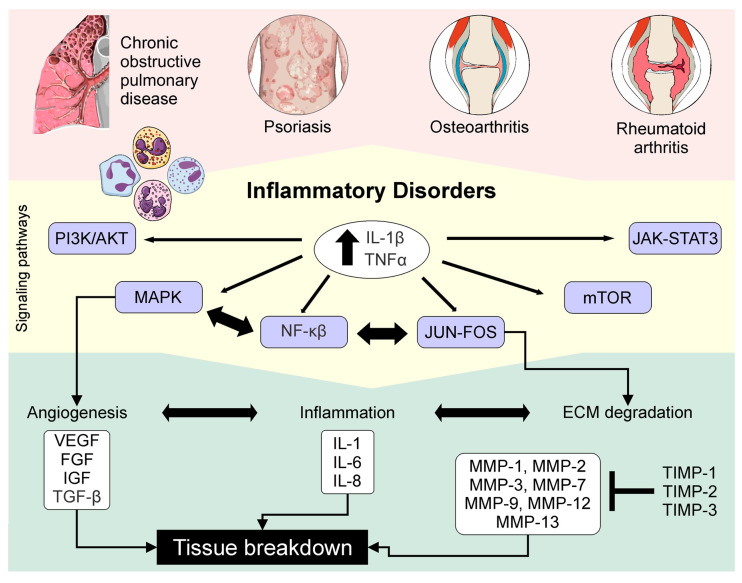
Roles of MMPs and TIMPs in the pathogenesis of human inflammatory diseases. Abnormal regulation and expression of MMPs and TIMPs within specific tissues is related to the development of inflammatory-related diseases such as rheumatoid arthritis, osteoarthritis, psoriasis, and chronic obstructive pulmonary disease. At the cellular level, the transcription of MMPs and TIMPs is regulated by growth factors, cytokines, and cell–extracellular matrix (ECM) interactions that can induce the activation of signaling pathways, including phosphoinositide 3-kinase/protein kinase B (PI3K/AKT), mitogen-activated protein kinase (MAPK), nuclear factor kappa-light-chain-enhancer of activated B cells (NF-κB), jun proto-oncogene–fos proto-oncogene/activator protein 1 (Jun-Fos/AP-1), mechanistic target of rapamycin kinase (mTOR), and the janus kinase–signal transducer and activator of transcription (JAK-STAT) pathway. Their abnormal activation may generate tissue breakdown. Some elements of this figure were taken from the Mind the Graph platform, available at www.mindthegraph.com. ↑ Means overexpressed.

Inflammation is a hallmark of many chronic and autoimmune diseases, including cancer, rheumatoid arthritis (RA), osteoarthritis (OA), psoriasis, and chronic obstructive pulmonary disease (COPD), among others. MMPs and TIMPs play key roles in the self-regulation of inflammation in these diseases [399,400] (Figure 4). OA is the most prevalent arthritic disease affecting the joints. It is a degenerative disease characterized by chronic joint pain, inflammation, and damage to the joint cartilage [401]. The articular cartilage is made up of type II collagen and proteoglycan aggregates. The integrity of the joint structure is dependent on these aggregates. MMP-13 is expressed by chondrocytes in human osteoarthritic cartilage, and it plays a key role in the degradation of type II collagen in bone and cartilage [402]. In patients with OA, variable levels of MMP-13 mRNA have been observed in osteoarthritic cartilage samples. The expression of both MMP-13 and MMP-1 in cartilage was significantly induced at both the mRNA and protein levels by interleukin-1a. Recombinant MMP-13 cleaved type II collagen, and MMP-13 turned over type II collagen at least 10 times faster than MMP-1 [403]. In OA models using samples of cartilage and human primary chondrocytes stimulated with IL-1β and TNF-α, the expression level of MMP-13 associated with degeneration in the joints and various inflammatory cytokines was measured. The involvement of MMP-13 in the pathogenesis of osteoarthritis was demonstrated by altering the physiological and anatomical functions of the joints [401,404]. The Akt signaling pathway and the activation of NF-κB signaling in chondrocytes are involved in the development of OA [405]. In OA, mononuclear cells (e.g., macrophages and T-cells) infiltrate the synovial membrane, and the levels of pro-inflammatory cytokines in synovial fluid and peripheral blood samples are elevated. Increased release of inflammatory mediators including IL-1β, IL-6, IL-8, IL-15, and TNF-α induces the expression of proteolytic enzymes such as MMPs, resulting in cartilage breakdown [406].

RA is an autoimmune disease of connective tissue that is characterized by destruction of the joint cartilage and, subsequently, of the under-lying bone [407]. MMPs and TIMPs have significant roles in ECM degradation associated with tissue damage in inflammation and RA. In vivo studies have shown elevated expression of MMP-2, MMP-3, and MMP-9 in RA. These enzymes break down the components of the non-collagenous matrix of the joints [408]. MMP-1 and MMP-13 have predominant roles in RA because they limit the rate of the collagen degradation process. In addition to collagen, MMP-13 degrades aggrecan and proteoglycan, giving it a dual role in ECM destruction in RA [408]. In RA, TIMPs are abundantly expressed in inflamed synovia. Synovial fluid samples from patients with RA were assessed for their reactivity with recombinant TIMPs. The presence of antibodies was analyzed, and the results were compared with healthy subjects. It was found that RA patients developed an autoimmune response to TIMPs (TIMP-1, TIMP-2) [409]. TIMP-1 and TIMP-2 retard plaque initiation and the progression of early lesions [242]. In vivo studies have shown that the over-expression of TIMP-3 decreases the plaque size and increases features of stability such as an increased collagen content and decreased necrotic core size in RA [410].

Psoriasis is a chronic skin condition driven by the activated immune system [411]. In this disease, MMPs are involved in the structural changes of the epidermis through the modification of intracellular contacts and the composition of the ECM, promoting angiogenesis in the dermal blood vessels and the infiltration of immune cells. Studies have shown that MMP-13 and MMP-9 participate in keratinocyte migration, angiogenesis, and contraction in wound healing in murine models [412]. In psoriasis, TIMP-1 and TIMP-3 are present in the inflammatory infiltrate and in the endothelial cells of the papillary dermis. TIMP-3 is downregulated at both transcriptional and protein levels in the epidermal keratinocytes associated with psoriatic lesions compared with respective healthy controls [413]. The levels of the TIMP1 protein in the blood serum increase after the occurrence of a psoriasis rash to a degree that correlates with the severity of the disease [414].

Another example of protease−antiprotease imbalance in the development of inflammatory disease occurs in COPD, which is a group of progressive lung diseases, most commonly emphysema and chronic bronchitis [415]. In a previous study, Uysal et al. classified patients with COPD by the severity of symptoms and risk of exacerbation, and they found that elevated plasma levels of MMP-9, TIMP-1, and TIMP-2 correlated with the severity of disease. The MMP-9 concentration and the MMP-9/TIMP-1 ratio were higher in patients with emphysema than in those with other phenotypes. This and other studies have suggested that the MMP-9 concentration and the MMP-9/TIMP-1 ratio are the best predictors of emphysema in COPD patients [415].

In summary, an imbalance of pro-inflammatory and anti-inflammatory cytokines contributes to the pathogenesis of RA, OA, psoriasis, and COPD through the dysregulation of MMPs and TIMPs (proteolytic axis). Inflammatory cytokines such as interleukin-1 beta (IL-1*β*) and TNFα can induce the activation of signaling pathways such as the nuclear factor kappa-light-chain-enhancer of activated B cells (NF-*κ*B), phosphoinositide 3-kinase/protein kinase B (PI3K/AKT), mitogen-activated protein kinase (MAPK), and AP-1 [416,417,418,419]. These pathways affect the expression of MMPs and TIMPs in inflammatory disorders (Figure 4). TNF-α induces the synthesis and secretion of MMPs which, in turn, affects cytokine and chemokine action. MMP-2, MMP-3, MMP-7, and MMP-9 release TGF*β* from the matrix [420]. Angiogenesis also occurs at the site of inflammation. MMPs also regulate the bioavailability of several angiogenic factors such as VEGF, fibroblast growth factor receptor (FGFR), epithelial growth factor, insulin-like growth factor, and TGF-β. At the cellular level, the transcription of MMPs and TIMPs is regulated by growth factors, cytokines, and cell–ECM interactions. The modulation of these pathways could provide protection against tissue destruction.

## 5. Targeting MMP and TIMP Function (Inhibitors)

The biology of MMPs and the understanding of their regulation led to the development of basic and preclinical research in animal models and clinical research in patients with pathologies including neurological, cardiovascular, gynecological, and inflammatory conditions—mainly cancer. These studies have focused on MMPs, because it is believed that they are the cause of alterations to important physiological mechanisms that lead to abnormal angiogenesis, apoptosis, and immune modulation, therefore contributing to tumor growth and/or metastasis [79]. Research studies funded by the pharmacological industry have begun with the development of broad-spectrum MMP inhibitors, but the precise physiology and biology of MMPs and the details of their evolution through time and regulation by other molecules are unknown. Clinical trials have not had the expected results, worsening the prognosis of diseases, as they were administered in advanced stages, causing adverse effects, such as musculoskeletal toxicity [79]. Nowadays, MMPs are known to have roles in physiological processes such as embryogenesis, angiogenesis, tissue remodeling, bone development, wound healing, mammary involution, cell migration, facilitation of the release of bound signaling molecules, activation of signaling molecules, and immunity [79]. MMP inhibitors and therapies that act against MMPs are listed below.

(a) Hydroxamate-based inhibitors. These molecules were the first MMP inhibitors to be developed. They are based on the structure of collagen and predominantly involve compounds containing a backbone designed to mimic the natural peptide substrate of the desired MMPs and a group that chelates the catalytic Zn2+ ion. They reduce the contribution of the rest of the compound to the inhibitor−enzyme binding process, thereby favoring broad-spectrum inhibition. Some examples of this type of drug are marimastat, ilomastat, and batimastat. Batimastat was the first MMP inhibitor to enter clinical trials, and it was shown to inhibit several MMPs, including MMP-1, MMP-2, MMP-7, and MMP-9, demonstrating antitumor effects in animal models of human ovarian cancer, colorectal cancer, melanoma, and hemangioma. Marimastat was found to be ineffective, and it caused musculoskeletal toxicity in a randomized Phase III trial for metastatic breast cancer cases that were stable or responding after first-line chemotherapy. The cause of this musculoskeletal pain is now thought to be the inhibition of ADAM and ADAMTS family members, including ADAM17. MMP inhibitors have often been administered too late to make a difference, as basic research was done in the early stages of the disease, and clinical research was intended to be a last aid in the treatment of terminal patients [79].

(b) New generation of hydroxamate-based MMP inhibitors. This new generation of MMP inhibitors has been made more specific, in an effort to decrease the adverse effects. Examples of them are MMI-270, MMI-166, PD-166793, ABT-770, cipemastat, and prinomastat. MMI-166 is a selective inhibitor of MMP-2, MMP-9, and MMP-14. PD-166793, prinomastat, and ABT-770 were developed to avoid binding to the “shallow pocket” of MMP-1 based on the idea (at that time) that MMP-1-sparing inhibitors would not induce musculoskeletal toxicity. Cipemastat, which inhibits MMP-1, MMP-3, and MMP-9, was used for the treatment of rheumatoid arthritis and osteoarthritis, but it was not shown to prevent the progression of joint damage in patients. New hydroxamate inhibitors are being developed using structure−activity relationship (SAR) analysis, which can aid in the identification of molecular substructures related to the presence or absence of biological activity [79].

(c) Non-hydroxamate MMP inhibitors. Hydroxamic acids are often metabolically labile, but there are several other zinc-binding groups that are stable. Reverse hydroxamates and non-hydroxamate inhibitors, for example, carboxylates, hydrocarboxylates, sulfhydryls, phosphoric acid derivatives, and hydantoins, were developed to avoid the limitations associated with first-generation MMP inhibitors, such as metabolic inactivation and the chelation of metals of other metalloproteins [79]. The MMP structure has been revealed with the use of crystallography, and new inhibitors have been designed with various peptidomimetic and non-peptidomimetic backbone structures. Rebimastat, a thiol zinc-binding group, is a broad-spectrum MMP inhibitor; however, a Phase II trial in early-stage breast cancer and a Phase III trial in non-small cell lung carcinoma both revealed adverse effects. [79]. Tanomastat was demonstrated to have difficulties with the dosing and timing of administration in relation to disease progression, and the efficacy of this drug was shown to be variable, with contradictory outcomes being obtained depending on the timing of administration [79]. Ro 28-2653 inhibits MT1-MMP, MT3-MMP, MMP-2, MMP-8, and MMP-9 but spares MMP-1 and ADAM17 activity. Several promising animal studies have demonstrated its antitumor and antiangiogenic activity, but they did not progress to clinical trials [79]. Potent inhibitors of MMP-13 and MMP-12 were developed using the alternative zinc-binding group hydantoin. Several biphenyl sulfonamide carboxylate MMP inhibitors were designed to treat osteoarthritis by inhibiting MMP-13. Tetracycline antibiotics, such as doxycycline and minocycline, have innate MMP inhibitory capacity. Doxycycline is indicated for use in periodontal disease and is the only collagenase inhibitor approved by the US Food and Drug Administration for any human disease [79].

(d) Targeting alternative binding sites. To reduce the off-target effects observed in clinical trials and to avoid broad MMP inhibition owing to the high structural homology of the different MMPs, recent research has switched from targeting the catalytic site to targeting alternative, less conserved sites. In addition to the Zn2+ ion in their catalytic sites, MMPs possess subsites (S) designated as unprimed or primed. The P1′−S1′ interaction is the main determinant of the affinity of inhibitors and cleavage positions of peptide substrates. For example, extension of the P1 substituent was used to gain MMP-13 selectivity over the highly homologous MMP-2. The use of NMR and X-ray crystallography methods combined with computational methods enables the modeling of drug−protein interactions with non-hydroxamate inhibitors of MMPs that bind to sites other than the catalytic sites. Several of these inhibitors demonstrated impressive MMP-13 selectivity and resulted in reduced clinical symptoms when tested in mouse models of rheumatoid osteoarthritis and arthritis. The combination of methods with computational prediction revealed hidden sites in the MMP structure, which can be exploited for the rational design of novel molecular effectors and therapeutic agents [79].

(e) Antibody-based therapeutics. These molecules have high selectivity and they possess several functional blocking antibodies. They selectively target the membrane-anchored MMPs. The highly selective antibody-based MMP-14 inhibitor DX-2400 has shown antitumor, antiangiogenic, and anti-invasive properties, and it blocks MMP-14-dependent pro-MMP-2 processing. MMP-14 inhibitory antibodies have been successfully tested in vitro and in vivo. Based on the three-dimensional structure and amino acid sequence of MMP-13, a neutralizing antibody that binds to the active form of MMP-13 but not to the latent form or to other MMPs was developed [79].

(f) Endogenous inhibitors of MMP function. α2-macroglobulin is a large serum protein that regulates MMP activity. MMPs are entrapped within the macroglobulin, thus preventing the MMPs from accessing large substrates. Polymyxin B-conjugated α2-macroglobulin demonstrated protective effects in mouse models of sepsis, and this effect was linked to its binding to, and neutralization of, inflammatory cytokines; MMP inhibition has not been studied [79].

TIMPs could theoretically form the basis of another novel class of MMP inhibitors, and they have rarely been considered. They have been used in model systems to yield vital clues about the efficacy of MMP inhibitors in diseases. However, TIMPs not only inhibit MMPs but, in specific cases, they can even indirectly promote MMP activity. For example, domain-specific overexpression of TIMP-2 and TIMP-3 revealed the MMP-independent functions of TIMPs during development [79].

Although TIMPs have been considered in current therapeutic strategies for the regulation of MMPs in different diseases, no success or progress has been achieved in this area, which is possibly due to the fact that some diseases share similar pathways or mechanisms of action where MMPs and TIMPs intervene and they do not play the exact same role. Common processes among the different diseases mentioned include inflammation, angiogenesis, cell death, and migration, among others. Our understanding of the subtleties of MMP biology is inadequate, so it is necessary to consider key points to increase knowledge in this field of study.

## 6. Concluding Remarks

To date, no accurate therapy based on MMP inhibition has been made available for clinical use in chronic diseases, so new strategies are needed to make proper use of the benefits provided by the known mechanisms of MMPs and TIMPs. The inhibition of MMP secretion and/or function in the correct place with the purpose of providing a better quality of life to those patients who have an illness related to the deregulation of MMPs may have advantages. It is necessary to continue basic research to explore the roles of MMPs involved in most of the diseases mentioned in this review, as has been the case for MMP-2 and MMP-9. It is known that the functions of MMPs are difficult to predict; however, by knowing the mechanisms of action by which they contribute to the aggravation of diseases, they can have benefits, not only as therapies but also as biomarkers and risk predictors of disease. According to the present review, it is clear that there is great heterogeneity in the status of TIMPs depending on the different diseases, because these differ in the types of interactions that they undergo with the distinct MMPs, as well as independently of MMPs. However, a generalized increase in TIMP-1 and a generalized silencing of TIMP-3 can be observed as disease progresses and the prognosis worsens. Additional studies are necessary for the specific investigation of these endogenous inhibitors in different chronic diseases. An alternative approach to the therapy of MMP-related diseases could be to use engineered TIMPs with restricted inhibitory specificities. It is necessary to broaden our knowledge on the specific roles of MMPs and TIMPs through studies and analyses focused on evaluating the activity of MMPs at the post-translational level, since most studies in the literature have only evaluated transcriptional activity, and there is little information on the intracellular activity of MMPs to rule out relevant functions of pathological importance that could provide more information on the different roles of MMPs and TIMPs in the different diseases mentioned in this review. It is also necessary to conduct more in vivo studies to demonstrate the functional activity of MMPs and TIMPs because it is not yet possible to elucidate how they are regulated/deregulated and to determine which substrates interfere in the different processes. Additional analyses are required to assess MMP activity under pathological and non-pathological conditions. We consider it necessary to emphasize the aforementioned points in order to carry out future research that will allow us to expand our knowledge and delve deeper into this field of study.

## Figures and Tables

**Table 1:** Classification of matrix metalloproteinases according with their structure and on the basis of their specificity for extracellular matrix components [6,7].

Feature	Subgroup	Description	MMPs
Structure	Minimal domain MMPs	Secreted	MMP-7, MMP-26
	Simple Hemopexin domain-containing MMPs	Secreted	MMP-1, -3, -8, 10, -12, -13, -18, -19, -22, -20, 27
	Gelating binding MMPs	Secreted	MMP-2, MMP-9
	Furin-activated MMPs	Secreted	MMP-11, MMP-28
	Vitronectin-like insert MMPs	Secreted	MMP-21
	Transmembrane MMPs (with cytoplasmic domain)	Membrane-type MMPs	TM: MMP-14, -15, -16, -24.
	glycosylphosphatidylinositol (GPI)-anchored MMPs	Membrane-type MMPs	MMP-17, MMP-25
	Type II transmembrane MMPs (cysteine array and immunoglobulin-like domains)	Membrane-type MMPs	MMP-23
Substrate	Collagenases (1–4)	Substrates: Col II, II, III, VIII, X, gelatins, aggrecan, entactin	MMP-1, -8,-13, -18
	Gelatinases (A and B)	Substrates: gelatins, collagens I, IV, V, VII, X, XI, fibronectin elastin, laminin, vitronectin, proMMPs-9 and 13	MMP-2, MMP-9
	Stromelysins (1–3)	Substrates: proteoglycans, laminin, gelatins, fibronectin, entactin, collagens III, IV, V, IX, X, XI, proMMPs 1, 8, 9 and 13, vitronectin, α 1-proteinase inhibitor	MMP-3, -10, -11
	Matrilysin	Substrates: proteoglycans, laminin, fibronectin, gelatins, entactin, collagen IV, elastin, tenascin	MMP-7, MMP-26
	Membrane-type MMPs (MT-MMP-1, -3, -4)	Substrates: col I, II, III, gelatins, fibronectin, laminin, proteoglycans, proMMP-2 and 13	MMP-14,-15, -16, -17, -24, -25

**Table 2:** Expression of metalloproteinases and tissue inhibitors of MMPs (TIMPs) during human embryonic development.

Action Site	MMPs or TIMPs	Function	Secretory Cell	Reference
Lung	MMP-1, MMP-9, TIMP-1-3	Branching Morphogenesis	Fetal epithelium	[11]
	MMP-1, MMP-2, TIMP-2, TIMP-3		Pulmonary vascular endothelium and media.	
	MMP-1, MMP-9	Angiogenesis		
	MMP-14	Alveolarization		
Hair (Lanugo)	MMP-2, MMP-9	Degrade gelatin, collagen IV, V, VII and X, fibronectin, and elastin.		[12,13,14]
	TIMP-2	Inhibits MMP-2		
	TIMP-1	Inhibits MMP-9		
Adipocyte	↓MMP-2, MMP-9	Adipocyte Clustering	Adipose Tissue	[15,16]
	↑MMP-3	Slow down adipogenic process		
	↑MMP-2, ↑MMP-9 ↓TIMP-1	Adipocyte differentiation		
	↑TIMP-1	Involutional adipogenesis		
Bone	↑MMP-2	Bone mineralization		[17,18]
	MMP-9	Implantation and bone resorption, bending strength and toughness of bone	Trophoblasts and osteoclasts	
	MMP-14	Membrane-bound protein	Peri-skeletal and skeletal tissue	
	MMP-16	Promoting bone growth and development	Osteoblasts and osteocytes	
	↑TIMP-1	Regulatory role in bone formation and remodeling. Stimulate the bone-resorbing activity of osteoclasts	Chondrocytes, osteoblasts, osteocytes, and osteoclasts.	
	↑TIMP-2	Stimulate the bone-resorbing activity of osteoclasts	Hypertrophic chondrocytes and osteoblasts	
Cartilage	MMP-13	Degradation of type II collagen	Hypertrophic cartilage during endochondral ossification	[19]
Membranes	MMP-8	Membrane rupture (birth)	Polymorphonuclear cells	[20]
	MMP-13		Amnion	
	↑MMP-2		Amnion	
	MMP-9		Amnion, trophoblast and decidual cells	
	MMP-3			
	MMP-2	Protection	Amnion	[21]

↑ Means overexpressed; ↓ Means underexpressed.

**Table 3:** Expression of metalloproteinases in different tissues.

MMP	Normal Expression (Tissues)	Expression in Human Diseases (Overexpression)	Reference
MMP-1	Kidneys, liver, colon, placenta, small intestines, stomach, bladder, and pancreas	Skin, heart, colon, lungs, pancreas, and umbilical cord	[29,30,31,32,33,34,35]
MMP-2	Lymph nodes, heart, adrenals, kidneys, liver, small intestines, colon, esophagus, lungs, ovaries, placenta, breasts, uterus, prostate, skin, bladder, testes, and CNS	Skin, kidneys, brain, heart, endometrium, bone marrow, ovaries, breasts, central nervous system (CNS), lungs, pancreas, circulatory system, and stomach	[29,36,37,38,39,40,41,42,43,44,45]
MMP-3	Tibial nerve, skeletal muscle, small intestine, colon adipocyte, salivary glands, stomach, bladder, and breasts	Kidney, brain, endometrium, colon, and pancreas	[35,36,41,46,47]
MMP-7	Kidneys, lung, bladder, pancreas, prostate, salivary gland, breasts, uterus, ovaries, and testis.	Blood vessels, heart, and pancreas	[48,49,50]
MMP-8	Bone marrow, lungs, and spleen	Skin, colon, and stomach	[51,52,53]
MMP-9	Bone marrow, colon, duodenum, lymph nodes, lungs, adrenals, ovaries, breasts, small intestine, placenta spleen, stomach, CNS, bladder, and kidneys	Skin, kidneys, brain, circulatory system, heart, endometrium, ovaries, colon, breasts, lungs, prostate, cardiovascular system, pancreas, and stomach	[29,32,36,41,42,44,45,48,51,54,55,56,57]
MMP-13	Lung, skin, prostate, small intestine, breast, testis, and bladder	Kidneys, colon, lung, and stomach	[32,36,58,59]
MMP-14	Adrenals, colon, uterus, esophagus, bladder, heart, brain, liver, kidneys, spleen, lungs, lymph node, prostate, skin, small intestine, stomach, breast, ovaries, and testes	Kidneys and brain	[36,60]
TIMP-1	Lymph nodes, brain, heart, arteries, colon, kidneys, liver, lungs, bladder, breasts, skin, ovaries, uterus, prostate, and testis	Breasts, brain, lungs, liver, kidneys, placenta, colon, circulatory system, skin, ovaries, and heart (overexpression)	[61,62,63,64,65,66,67]
TIMP-2	Lymph nodes, brain, heart, arteries, colon, kidneys, liver, breast, ovaries, prostate, and testes	Kidneys, brain, heart, and ovaries, bone marrow and amniotic fluid (over- and underexpression)	[37,39,65,68]
TIMP-3	Brain, heart, colon, kidneys, lungs, liver, breast, ovaries, prostate and testes, and eyes	Kidneys, liver, brain, heart, ovaries, cardiovascular system lungs, placenta, and breasts (underexpression)	[69,70,71,72,73]
TIMP-4	Brain, heart, breast, uterus, ovary, kidney, pancreas, colon, testes, adipose tissue and prostate	Heart, brain and uterus, cardiovascular system and circulatory system (over- and underexpression)	[63,74,75,76,77]

## Supplementary Materials

Supplementary Materials can be found at https://www.mdpi.com/1422-0067/21/24/9739/s1.

## Abbreviations

MMPs	Matrix metalloproteinases
TIMPs	Tissue inhibitors of metalloproteinases
ECM	Extracellular matrix
MT-MMPs	Membrane type matrix metalloproteases
GPI	Glycosylphosphatidylinositol
Ig	Immunoglobulin
TM	Transmembrane domain
ADAM	Disintegrin and metalloproteinase
WG	Weeks of gestation
WAT	White adipose tissue
BAT	Brown adipose tissue
DM	Diabetes mellitus
EVs	Extracellular vesicles
T2DM	Type 2 DM
GDM	Gestational diabetes mellitus
miRs	microRNAs
CVD	Cardiovascular disease
DFU	Diabetic foot ulcer
CKD	Kidney disease
GBM	Glomerular basement membrane
AKI	Acute kidney injury
CKD	Chronic kidney disease
CNS	Central nervous system
AD	Alzheimer’s disease
MS	Multiple sclerosis
BBB	Blood−brain barrier
PD	Parkinson’s disease
MHC	Major histocompatibility complex
MAPK	Mitogen-activated protein kinases
TNF-αALS	Tumor necrosis factor alphaAmyotrophic lateral sclerosis
CSF	Cerebrospinal fluid
APP	Amyloid precursor protein
CVD	Cardiovascular disease
TOD	Target organ damage
MI	Myocardial infarction
PCOS	Polycystic ovarian syndrome
SA	Spontaneous abortion
RPL	Recurrent pregnancy loss
PE	Preeclampsia
VEGF	Vascular endothelial growth factor
GCM1	Factor glial cell missing-1
BMDCs	Bone-marrow-derived cells
IL	Interleukin
HLA	Human leukocyte antigen
TGF-β	Transforming growth factor beta
ERK	Extracellular regulated kinase
FGF	Fibroblast growth factor
LOXL	Lysyl oxidase
RA	Rheumatoid arthritis
OA	Osteoarthritis
COPD	Chronic obstructive pulmonary disease
HF	Heart failure
